# Site-Specific Fluorogenic Protein Labelling Agent for Bioconjugation

**DOI:** 10.3390/biom10030369

**Published:** 2020-02-28

**Authors:** Kelvin K. Tsao, Ann C. Lee, Karl É. Racine, Jeffrey W. Keillor

**Affiliations:** Department of Chemistry and Biomolecular Sciences, University of Ottawa, Ottawa, ON K1N 6N5, Canada; ktsao072@uottawa.ca (K.K.T.); alee189@uottawa.ca (A.C.L.); kraci097@uottawa.ca (K.É.R.)

**Keywords:** protein labelling, heterobifunctional, fluorogenic, CuAAC

## Abstract

Many clinically relevant therapeutic agents are formed from the conjugation of small molecules to biomolecules through conjugating linkers. In this study, two novel conjugating linkers were prepared, comprising a central coumarin core, functionalized with a dimaleimide moiety at one end and a terminal alkyne at the other. In our first design, we developed a protein labelling method that site-specifically introduces an alkyne functional group to a dicysteine target peptide tag that was genetically fused to a protein of interest. This method allows for the subsequent attachment of azide-functionalized cargo in the facile synthesis of novel protein-cargo conjugates. However, the fluorogenic aspect of the reaction between the linker and the target peptide was less than we desired. To address this shortcoming, a second linker reagent was prepared. This new design also allowed for the site-specific introduction of an alkyne functional group onto the target peptide, but in a highly fluorogenic and rapid manner. The site-specific addition of an alkyne group to a protein of interest was thus monitored in situ by fluorescence increase, prior to the attachment of azide-functionalized cargo. Finally, we also demonstrated that the cargo can also be attached first, in an azide/alkyne cycloaddition reaction, prior to fluorogenic conjugation with the target peptide-fused protein.

## 1. Introduction

A number of clinically relevant therapeutic agents have been formed from the covalent attachment of synthetic polymers, drugs, and imaging probes to proteins or antibodies [1,2]. These protein-polymer or antibody-drug conjugates are formed through a number of linking strategies that connect the biomolecule to cargo. Appending cargo to these biomolecules relies on highly efficient and bioorthogonal “click” type reactions such as Michael addition, copper-catalyzed azide-alkyne cycloaddition (CuAAC), strained-promoted azide-cycloalkyne cycloaddition (SPAAC) and the Staudinger ligation [3,4,5]. The link between the protein and the cargo can be non-site specific, as in the case of modifying natural amino acids, or highly site specific, as is the case with labelling genetically fused tags or introducing unnatural amino acids [6,7]. Both the reactions used and the degree of site-specificity of the conjugation must be considered and optimized in order to effectively build new protein conjugates.

Bioconjugates are most commonly synthesized by appending cargo to a protein or antibody through a linker that provides a site of attachment for the intended cargo. Examples of relevant cargo include polyethylene glycol [8,9,10,11], to improve pharmacokinetic properties, or a small molecule drug [12,13], for targeted delivery. Traditionally, the conjugation is monitored discontinuously (or ex situ), using methods such as sodium dodecyl sulfate-polyacrylamide gel electrophoresis (SDS PAGE), high throughput liquid chromatography (HPLC), or mass spectrometry. More recently developed linkers allow for the continuous (or in situ) monitoring of the bioconjugation event by fluorescence, due to their fluorogenic nature. Many of these linkers have been reviewed by Liu and coworkers, but three significant methods stand out [14]. Most recently, Liu reported a fluorogenic linker that allowed the attachment of cargo through CuAAC on one end of the linker and reaction with a target protein’s native Cys thiol group through a Michael addition on the other [15]. O’Reilly et al. presented the fluorogenic conjugation of PEG onto salmon calcitonin (sCT) through an *N*-propargyldibromomaleimide linker [16]. Lastly, Cornelissen and coworkers developed a technique that conjugated PEG cargo to azide functionalized bovine serum albumin (BSA) through a fluorogenic 3-azidocoumarin linker [17]. However, drawbacks of the above linkers include dependence on the reactivity of an intrinsic Cys residue, reversible conjugation or the requirement of specific techniques to introduce unnatural amino acids into the protein. Thus, there remains a need for complementary methods that will expand the scope of biomolecules that can be easily and efficiently conjugated.

The modular design of novel protein-cargo conjugates requires a linker that can be easily integrated into any protein. Furthermore, the conjugation method should also be site specific, so as not to disrupt the native function of the protein itself. Lastly, it would be advantageous for the linker to be fluorogenic such that the conjugation can be monitored in situ. Thus, we propose a fluorogenic linker that reacts site-specifically with a small peptide tag genetically fused to either terminus of a target protein. This approach eliminates the necessity of incorporating unnatural amino acid residues into a protein of interest, while also providing a specific site of modification that can be easily appended onto any protein with minimal disruption of that protein’s function.

In previous work, we have developed a fluorogenic specific protein labelling method based on the conjugation between a pro-fluorescent probe and a small α-helical peptide genetically fused to the protein of interest [18,19]. The fluorogenic probes (referred to as FlARe probes) consist of a fluorophore covalently attached to two maleimide groups that quench the fluorophore’s emission through a photoinduced electron transfer (PeT) mechanism [20,21]. These bismaleimide probes have also been shown to react highly efficiently with the two activated cysteine thiols, located 10 Å apart, on a rationally designed α-helical target peptide (dC10*) in a **Fl**uorogenic **A**ddition **Re**action (FlARe) [22,23]. When both maleimides react with the chemically tuned thiols, the PeT quenching is abolished, and the intrinsic fluorescence of the FlARe probe is restored.

**Scheme 1:**
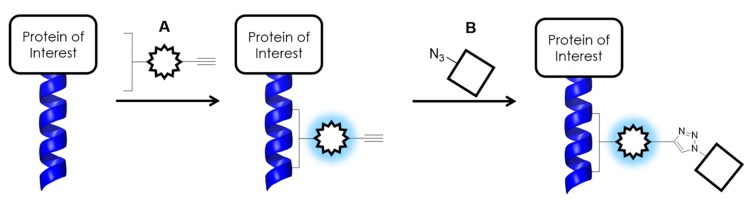
A protein-cargo conjugate is formed by first labelling a protein with a heterobifunctional fluorogenic coumarin linker (**A**). Azide-functionalized cargo (**B**) is then attached to the protein through the alkyne moiety on the fluorogenic probe. Thus, fluorogenic and site-specific bioconjugation is achieved.

Herein, we report a method for the site-specific and fluorogenic introduction of an alkyne functional group onto a specific protein. This method uses a heterobifunctional pro-fluorescent linker that takes advantage of the FlARe protein labelling technology (Scheme 1). The proposed linker consists of a coumarin fluorophore core, functionalized on one end with a dimaleimide moiety. These maleimide functional groups quench the coumarin’s fluorescence and allow the linker to react with the dC10* tag that is genetically fused to a protein of interest. This coumarin FlARe probe is additionally functionalized with a terminal alkyne on the other end, such that it modifies a specific protein bearing the dC10* tag by site-specifically introducing an alkyne group onto the exposed terminal target peptide sequence. Since the reaction between the linker and the dC10* tag is fluorogenic, the initial conjugation can be monitored in situ by fluorescence increase. After the initial labelling reaction is complete, the modified protein bearing the alkyne functional group can then be conjugated to a broad array of azide-functionalized cargo through CuAAC. Overall, this novel linker allows for a facile and effective method for the in vitro synthesis of fluorescent protein-conjugates that is easily amenable to engineered proteins of therapeutic significance.

## 2. Materials and Methods

### 2.1. Expression and Purification of Test Protein

The plasmid coding for the tagged test protein maltose binding protein (MBP-dC10*) was designed and created in a previously published study in which the reactivity of the original dC10 tag was improved through the introduction of positively charged residues around the cysteines [22]. The expression and purification of MBP-dC10* followed the protocol from the same study and consisted of transforming the expression plasmid into the BL21-Gold (DE3) strain of *E. coli* cells [22]. Expression of MBP-dC10* was induced with IPTG (0.3 mM) and purification was performed an amylose resin column and elution with 10 mM maltose. According to the Bradford Assay, protein yields generally ranged around 12 mg from 500 mL of expression culture.

### 2.2. Determination of Fluorescence Enhancement Ratios

Emission spectra and fluorescence intensity measurements were recorded at 25 °C with a Synergy H4 Hybrid Multi-Mode Microplate Reader with excitation and emission monochromators set at 9-nm bandpass. The initial excitation and emission spectra were recorded for a solution of 10 µM labelling reagent (compounds **1**, **6**, **10**, or **15**) in 50 mM HEPES (I = 100 mM with NaCl, pH 7.4) with 10% DMSO and 50 µM TCEP. A solution of 10 µM labelling reagent in 50 mM HEPES (I = 100 mM with NaCl, pH 7.4) with 10% DMSO and 50 µM TCEP with the addition of 10 µM MBP-dC10* was allowed to react for 4 h (in the case of **1** or **15**) or 24 h (in the case of **6** or **10**). After the reaction was complete, the final excitation and emission spectra were recorded. The ratio of fluorescence intensity at the maximum emission between the initial and final spectra gave the fluorescence enhancement (FE).

### 2.3. Kinetic Studies

Kinetic experiments were carried out at 25 °C using a Synergy H4 Hybrid Multi-Mode Microplate Reader with excitation and emission monochromators set at a 20-nm bandpass. Solutions containing 10 µM MBP-dC10* in 50 mM HEPES buffer (I = 100 mM, pH 7.4) with 10% DMSO and 50 µM TCEP were prepared in a 96-well plate. Labelling reagent was added immediately before recording to a final concentration of 10 µM. Samples were excited at 435 nm for **10** and **15** or 415 nm for **1** and fluorescence intensity was followed at 485 nm for **10** and **15** or 460 nm for **1** as a function of time. All time-dependent fluorescence curves were fitted to a second order rate equation to obtain the second order rate constant (*k*_2_) for the reaction between the label and MBP-dC10*.

### 2.4. Bioconjugation of Rhodamine B Azide to MBP-dC10*-**15** or **6** Adduct

The protocol for performing the CuAAC between rhodamine B azide (RhB-N_3_) and MBP-dC10* labelled with **6** or **15** was adapted from procedures developed by Nasheri, et al. and Yang, et al. [24,25]. An aliquot of 5 µM **15** was incubated with 2.5 µM MBP-dC10* and 50 µM TCEP in HEPES buffer (50 mM HEPES (I = 100 mM NaCl), pH 7.4) at 25 °C and a total volume of 200 µL for 1 h. The reaction was monitored using a Synergy H4 Hybrid Multi-Mode Microplate Reader with excitation (435 nm) and emission (485 nm) monochromators set at a 20-nm bandpass. After complete reaction, the mixture was transferred to a 500-µL 10-kDa MWCO Amicon filter and buffer exchanged into fresh HEPES buffer three times. After the third time, the solution was adjusted to 100 µL. A 500-µL click mixture (1.0 mM CuSO_4_, 100 µM TBTA, 1.0 mM TCEP-HCl, 50 µM RhB-N_3_, PBS pH 7.4, 0.25% DMSO) was prepared and 100 µL of this click mixture was added to the buffer exchanged solution containing the MBP-dC10*-**15** adduct. The click reaction was allowed to progress for 2 h in the dark with gentle shaking at 25 °C. After this, 500 µL of cold acetone (chilled to −80 °C) was added and the protein was precipitated overnight at -80 °C. The protein was pelleted by spinning at 14,000 rpm for 15 min at 4 °C before removing the supernatant and drying off any excess acetone under a gentle stream of air. After, the pellet was washed with 100 µL MeOH chilled to −80 °C before pelleting again at 14,000 rpm for 15 min at 4 °C. The precipitate was then taken up in 35 µL SDS PAGE loading buffer (50 mM TCEP) and shaken for 15 min at room temperature. The samples were spun at 14,000 rpm for 5 min and then boiled at 90 °C for 5 min before 10 µL was loaded onto a 10%-acrylamide SDS-PAGE gel and run for 1.5 h at 120 V.

In the case of **6,** the reaction between MBP-dC10* and **6** was allowed to react for 24 h and the reaction was not monitored.

### 2.5. Bioconjugation of Rh-B-N_3_-**15** Adduct to MBP-dC10*

The protocol for performing the CuAAC between Rhodamine B azide (RhB-N_3_) and **15** was adapted from procedures developed by Nasheri, et al. and Yang, et al. [24,25]. A 500-µL click mixture (1.0 mM CuSO_4_, 100 µM TBTA, 1.0 mM TCEP-HCl, 50 µM RhB-N_3_, PBS pH 7.4, 0.25% DMSO) was first prepared and 90 µL of this click mixture was incubated with 10 µL of a 200 µM **15** stock (final concentration 20 µM). The click reaction was allowed to progress in the dark at r.t. for 2 h with gentle shaking. After, 100 µL of a 10-µM MBP-dC10* solution was added to the mixture and the entire solution was allowed to react for 1 h at r.t. Next, 500 µL of cold acetone (chilled to −80 °C) was added and the protein was precipitated overnight at −80 °C. The protein was pelleted by spinning at 14,000 rpm for 15 min at 4 °C before removing the supernatant and drying off any excess acetone under a gentle stream of air. The pellet was washed with 100 µL MeOH chilled to −80 °C before pelleting again at 14,000 rpm for 15 min at 4 °C. The precipitate was taken up in 35 µL SDS PAGE loading buffer (50 mM TCEP) and sonicated for 3 minutes prior to being shaken for 15 min at room temperature. The samples were spun at 14,000 rpm for 5 min and then boiled at 90 °C for 5 min before 5 µL was loaded onto an 10% acrylamide SDS-PAGE gel and run for 1.5 h at 120 V.

### 2.6. Synthesis

All reagents and solvents for reactions were used as received unless otherwise stated. Dichloromethane, dimethylformamide, methanol and acetonitrile were dried using a solvent purification system from LC Technology Solutions Inc (Salisbury, USA).

Reactions were monitored by thin layer chromatography (TLC) using E. Merck silica gel 60F254 precoated aluminum plates. Components were visualized by illumination with a short-wavelength ultraviolet light or long-wavelength visible light. Flash column chromatography was performed on ZEOCHEM^®^ silica gel 60 (ECO 40–63 µm) using ethyl acetate/n-hexane or methanol/dichloromethane as eluting solvents.

Nuclear magnetic resonance (NMR) spectra were recorded in deuterated DMSO or deuterated chloroform at ambient temperature unless otherwise stated. The experiments were performed either on a Bruker Avance 400 Fourier Transform Spectrometer operating at 400 MHz for ^1^H and at 100 MHz for ^13^C or on a Bruker Avance 600 Fourier Transform Spectrometer operating at 600 MHz for ^1^H and at 150 MHz for ^13^C. EI-MS spectra were recorded on a Kratos Concept mass spectrometer for both low resolution and high-resolution mass spectra. ESI-MS spectra were recorded on a Waters Micromass Q-TOF mass spectrometer. Melting points were measured on an EZ-Melt automated melting point apparatus and are uncorrected. Ultraviolet absorbance spectra and fluorescence spectroscopic studies were performed on a Synergy H4 Hybrid Multi-Mode Microplate Reader.

**Compound 5**: Compound **5** was synthesized according to the procedures laid out by Chen and coworkers [19].

**Compounds 2** and **3** were synthesized according to the procedures laid out by Abdizadeh and coworkers [26].

**Compound 2**: To a solution of 4-hydroxysalicylic acid (1.38 g, 10.0 mmol) and diethylmalonate (1.0 eq, 1.53 mL, 10.0 mmol) in EtOH (5 mL) was added piperidine (0.02 eq, 19.8 µL, 0.2 mmol) and acetic acid (0.02 eq, 11.4 µL, 0.2 mmol). The solution was stirred at room temperature for 20 min and then heated to 78 °C and stirred for 3 h. The reaction was cooled to room temperature and the product was crystallized as an orange solid in 81% yield. ^1^H NMR (400 MHz, DMSO) δ 11.04 (s, 1H), 8.73–8.53 (m, 1H), 7.71 (d, *J* = 8.6 Hz, 1H), 6.80 (dd, *J* = 8.6, 2.3 Hz, 1H), 6.75–6.47 (m, 1H), 4.22 (q, *J* = 7.1 Hz, 2H), 1.25 (t, *J* = 7.1 Hz, 3H). ^13^C NMR (100 MHz, DMSO) δ 164.5, 163.4, 157.6, 156.9, 149.9, 132.6, 114.5, 112.6, 110.9, 102.3, 61.3, 14.6. HRMS (ESI): calcd for C_12_H_10_O_5_Na ([MNa]^+^): 257.0426, found: 257.0414.

**Compound 3**: Compound **2** (50 mg, 0.159 mmol) was dissolved in acetonitrile (3.0 mL), to which a solution of NaOH (10.0 eq, 63.6 mg, 1.59 mmol) dissolved in water (2.0 mL) was added dropwise. The solution was stirred at room temperature for 3.5 h after which it was diluted with 10 mL water and then acidified with 10 mL 1 M HCl. The aqueous solution was extracted with EtOAc (3 × 30 mL) and the combined organic layers were dried with MgSO_4_. After removing the solvent on the rotary evaporator, compound **3** was obtained as a yellow solid in quantitative yield. ^1^H NMR (400 MHz, DMSO) δ 12.00 (bs, 1H), 8.64 (s, 1H), 7.73 (d, *J* = 8.4 Hz, 1H), 6.83 (d, *J* = 8.4 Hz, 1H), 6.72 (s, 1H). ^13^C NMR (100 MHz, DMSO) δ 164.32 (d, *J* = 14.1 Hz), 157.75, 156.99, 148.96, 131.91, 114.17, 112.69, 110.53, 101.83. ^13^C NMR (100 MHz, DMSO) δ 164.3, 157.8, 157.0, 149.0, 131.9, 114.2, 112.7, 110.5, 101.8. HRMS (ESI): calcd for C_10_H_6_O_5_Na ([MNa]^+^): 229.0113, found: 229.0128.

**Compound 1**: To a solution of compound **3** (26.0 mg, 0.130 mmol) in anhydrous DMF (2.5 mL) was added HBTU (1.1 eq, 52.0 mg, 0.138 mmol). The mixture was stirred at room temperature for 10 min before **5** (1.5 eq, 59.0 mg, 0.188 mmol) was added. The mixture was stirred for an additional 20 min before trimethylamine (3.0 eq, 50 µL, 0.380 mmol) was added. It was then stirred overnight followed by diluting the mixture in EtOAc (100 mL) and washing with 1 M HCl (2 × 100 mL). The organic layer was dried with MgSO_4_ before the solvent was removed using a rotary evaporator. The crude was purified by flash chromatography using a gradient elution system of 0%–5% MeOH in DCM to obtain compound **1** as a pale-yellow solid in 7% yield. ^1^H NMR (600 MHz, DMSO) δ 11.18 (bs, 1H), 10.86 (s, 1H), 8.83 (s, 1H), 7.86 (d, *J* = 8.7 Hz, 1H), 7.78 (d, *J* = 1.7 Hz, 2H), 7.12 (t, *J* = 1.8 Hz, 1H), 6.92 (dd, *J* = 8.6, 2.2 Hz, 1H), 6.85 (d, *J* = 2.0 Hz, 1H), 6.83 (q, *J* = 1.8 Hz, 2H), 2.09 (d, *J* = 1.7 Hz, 6H). ^13^C NMR (150 MHz, DMSO) δ 170.3, 169.4, 164.1, 161.2, 160.7, 156.5, 149.6, 148.4, 145.9, 138.7, 132.6, 132.3, 127.6, 120.0, 116.8, 114.6, 114.1, 111.2, 102.0, 10.9. HRMS (ESI): calcd for C_26_H_17_N_3_O_8_Na ([MNa]^+^):522.0913, found: 522.0929. **mp**: decomp. at 267.7–271.1 °C.

**Compound 7**: The synthesis of compound **7** was done according to Jiang, et al. [27]. A Finkelstein reaction was performed to synthesize **7** in which 6-chloro-1-hexyne (8.30 mmol, 1.00 mL) was added to dry acetone (100 mL) along with NaI (58.1 mmol, 8.71 g, 7 eq). The solution was brought to reflux and stirred overnight after which the acetone was removed using a rotary evaporator. The residue was then partitioned between hexanes (100 mL) and water (100 mL) and the organic layer was washed with water (100 mL) and brine (100 mL). The organic layer was then dried with MgSO_4_ and the solvent was removed using a rotary evaporator to obtain compound **7** as a transparent liquid in 94% yield (7.78 mmol, 1.62 g). ^1^H NMR (400 MHz, CDCl_3_) δ ppm 3.20 (t, *J* = 6.91 Hz, 2 H), 2.22 (td, *J* = 6.98, 2.60 Hz, 3 H), 1.95 (m, 3 H), 1.63 (tt, *J* = 7.30 Hz, 3 H).

**Compound 8**: Compound **8** (1.00 mmol, 234 mg) was dissolved in acetonitrile after which compound **7** was added. K_2_CO_3_ (1.2 mmol, 165 mg, 1.2 eq) and a catalytic amount of 18-crown-6 (0.1 mmol, 26.4 mg) was then added to the reaction mixture, which was brought to reflux and stirred overnight. The acetonitrile was removed using a rotary evaporator and the residue was partitioned between EtOAc (20 mL) and water (20 mL). The aqueous layer was extracted with EtOAc (3 × 30 mL) and the combined organic layers were dried with MgSO_4_ and the solvent was removed. The residue was purified by flash chromatography using a gradient elution system of 10%–30% EtOAc in hexanes. Compound **8** was obtained as a white solid in 44% yield (0.437 mmol, 138 mg). ^1^H NMR (400 MHz, CDCl_3_) δ ppm 8.50 (s, 1 H), 7.49 (d, *J* = 8.72 Hz, 1 H), 6.88 (dd, *J* = 8.67, 2.20 Hz, 1 H), 6.80 (d, *J* = 2.06 Hz, 1 H), 4.40 (q, *J* = 7.15 Hz, 2 H), 4.08 (t, *J* = 6.22 Hz, 2 H), 2.30 (td, *J* = 6.91, 2.55 Hz, 2 H), 1.97 (m, 3 H), 1.74 (tt, *J* = 7.30 Hz, 2 H), 1.40 (t, *J* = 7.10 Hz, 3 H). ^13^C NMR (100 MHz, CDCl_3_) δ ppm 164.6, 163.5, 157.6, 157.2, 149.0, 130.7, 114.0, 114.0, 111.6, 100.8, 83.7, 68.9, 68.3, 61.7, 27.9, 24.9, 18.1, 14.3. HRMS (ESI): calcd for C_18_H_18_O_5_Na ([MNa]^+^): 337.1052, found: 337.1074. **mp**: 92.6–93.6 °C.

**Compound 9**: To a solution of **8** (0.437 mmol, 138 mg) dissolved in acetonitrile, NaOH (4.93 mmol, 197 mg, 10 eq) was added as a 2 M solution in water (2.5 mL). The reaction was stirred for 3.5 h after which it was partitioned between water (10 mL), 1 M HCl (10 mL) and EtOAc (30 mL). The aqueous phase was extracted with EtOAc (3 × 30 mL) and the combined organic phases were dried with MgSO_4_. The remaining EtOAc was removed using a rotary evaporator to obtain compound **9** as a white solid in 97% yield (0.425 mmol, 122 mg). ^1^H NMR (400 MHz, DMSO) δ ppm 12.97 (br. s., 1 H), 8.71 (s, 1 H), 7.82 (d, *J* = 8.72 Hz, 1 H), 7.01 (m, 2 H), 4.14 (t, *J* = 6.22 Hz, 2 H), 2.79 (t, *J* = 2.55 Hz, 1 H), 2.24 (td, *J* = 6.69, 2.20 Hz, 2 H), 1.83 (tt, *J* = 8.50, 7.70 Hz, 2 H), 1.61 (tt, *J* = 7.30 Hz, 2 H). ^13^C NMR (100 MHz, DMSO) δ ppm 164.1, 163.9, 157.2, 156.8, 148.9, 131.5, 113.7, 113.5, 111.5, 100.6, 84.1, 71.4, 68.0, 27.4, 24.4, 17.3. HRMS (ESI): calcd for C_16_H_14_O_5_Na ([MNa]^+^): 309.0739, found: 309.0727. **mp**: 170.0–170.9 °C.

**Compound 6**: Compound **9** (0.349 mmol, 100 mg) was dissolved in SOCl_2_ (6 mL). The solution was brought to reflux and stirred for 6 h before removing excess SOCl_2_ using the rotary evaporator. The residue was dissolved in pyridine (5 mL) to which compound **5** (0.349 mmol, 149 mg, 1.0 eq) dissolved in pyridine (5 mL) was added and the mixture was left to stir overnight. The solution was then taken up in EtOAc (20 mL) and then washed with 1 M HCl (3 × 20 mL), 5% CuSO_4_ (20 mL) and brine (20 mL). The organic layer was dried with MgSO_4_ and the solvent was removed on a rotary evaporator to obtain the crude residue, which was purified by flash chromatography using a 5:4:1 hexanes/DCM/EtOAc mixture. Compound **6** was obtained as a pale-yellow solid in 12% yield (0.0431 mmol, 25 mg). ^1^H NMR (400 MHz, CDCl_3_) δ ppm 10.96 (s, 1 H), 8.92 (s, 1 H), 7.85 (d, *J* = 1.86 Hz, 2 H), 7.62 (d, *J* = 8.72 Hz, 1 H), 7.28 (t, *J* = 1.86 Hz, 1 H), 6.96 (dd, *J* = 8.72, 2.35 Hz, 1 H), 6.89 (d, *J* = 2.25 Hz, 1 H), 6.50 (q, *J* = 1.67 Hz, 2 H), 4.11 (t, *J* = 6.27 Hz, 2 H), 2.31 (td, *J* = 6.96, 2.65 Hz, 2 H), 2.18 (d, *J* = 1.86 Hz, 6 H), 1.99 (m, 3 H), 1.75 (m, 2 H). ^13^C NMR (100 MHz, CDCl_3_) δ ppm 170.1, 168.9, 164.7, 162.1, 160.1, 156.9, 149.3, 145.9, 138.9, 132.7, 131.2, 127.6, 118.4, 116.4, 114.7, 114.3, 112.4, 100.8, 83.7, 69.0, 68.4, 27.9, 24.8, 18.1, 11.2. HRMS (ESI): calcd for C_32_H_25_N_3_O_8_Na ([MNa]^+^): 602.1539, found: 602.1550. **mp**: decomp. at 192.9–194.2 °C.

**Compound 12**: To a solution of 3-aminophenol (1 g, 9.2 mmol, 2.0 eq) in THF (20 mL) was added 4-ethynylbenzaldehyde. The solution was stirred for 1.5 h at r.t. prior to adding STAB (1.36 g, 6.44 mmol, 1.4 eq). After stirring for 3 h at r.t., the reaction was quenched with sat. NaHCO_3_ (60 mL) and extracted with ether (3 × 20 mL). Compound **12** was purified using flash chromatography with a 2:8 EtOAc/hexanes eluent and obtained as a transparent viscous oil in 75% yield. ^1^H NMR (400 MHz, CDCl_3_) δ ppm 7.51–7.39 (m, 2H), 7.31 (d, *J* = 8.4 Hz, 2H), 7.01 (t, *J* = 8.0 Hz, 1H), 6.20 (dddd, *J* = 10.4, 8.0, 2.3, 0.8 Hz, 2H), 6.08 (t, *J* = 2.3 Hz, 1H), 4.32 (s, 2H), 3.06 (s, 1H). ^13^C NMR (100 MHz, CDCl_3_) δ 156.8, 149.6, 140.4, 132.6, 130.4, 127.4, 121.1, 106.2, 104.9, 99.8, 83.6, 48.1. HRMS (EI): calcd for C_15_H_13_NO: 223.09971, found: 223.09831.

**Compound 13**: Compound **12** (460 mg, 2.06 mmol) was dissolved in MeOH and cooled to 0 °C. Once cooled, acetaldehyde (4.0 eq, 0.461 mL, 8.24 mmol) was added and the solution was stirred for 2 h. After, the solution was removed from ice and then NaBH_4_ (6.0 eq., 468 mg, 12.4 mmol) was added and the mixture stirred at room temperature overnight. Purification by flash chromatography by gradient elution (0%–30% EtOAc in hexane) yielded compound **13** in 50% yield as a transparent viscous oil. ^1^H NMR (400 MHz, CDCl_3_) δ ppm 7.43 (d, *J* = 8.23 Hz, 2 H), 7.18 (d, *J* = 7.84 Hz, 2 H), 7.02 (t, *J* = 8.28 Hz, 1 H), 6.25 (m, 1 H), 6.15 (m, 2 H), 4.62 (s, 1 H), 4.48 (s, 2 H), 3.43 (q, *J* = 7.09 Hz, 2 H), 3.04 (s, 1 H), 1.19 (t, *J* = 7.05 Hz, 3 H). ^13^C NMR (100 MHz, CDCl_3_) δppm 156.7, 149.9, 140.1, 132.4, 130.2, 126.5, 120.5, 105.1, 103.3, 99.2, 83.6, 53.8, 45.4, 12.2. HRMS (ESI): calcd for C_17_H_18_NO ([MH]^+^): 252.1388, found: 252.1362.

**Compound 11**: The synthesis of compound **4** was adapted from a procedure developed by Takakura, et al. [28]. POCl_3_ (0.464 mL, 4.77 mmol, 4.0 eq) was added dropwise to DMF (1.06 mL, 13.7 mmol, 1.5 eq.) over ice and stirred for 5 min. Compound **13** was then added and the solution was stirred at r.t. for 1 h. Afterwards, the solution was heated to 70 °C and stirred for 1 h before cooling the solution to r.t. Hydrolysis was performed by adding H_2_O (10 mL) to the reaction and stirring for 30 min. The solution was then neutralized with NaOH and extracted with EtOAc (3 × 50 mL). Compound **11** was purified by flash chromatography using a 2:8 EtOAc/hexanes elution system and was obtained as a white solid in 44% yield. ^1^H NMR (400 MHz, CDCl_3_) δ ppm 11.57 (s, 1H), 9.57–9.18 (m, 1H), 7.45 (d, *J* = 8.3 Hz, 2H), 7.26 (t, *J* = 4.4 Hz, 2H), 7.13 (d, *J* = 8.5 Hz, 2H), 6.26 (dd, *J* = 8.9, 2.4 Hz, 1H), 6.12 (d, *J* = 2.4 Hz, 1H), 4.59 (s, 2H), 3.52 (q, *J* = 7.1 Hz, 2H), 3.06 (s, 1H), 1.26 (t, *J* = 7.1 Hz, 3H). ^13^C NMR (100 MHz, CDCl_3_) δ 192.4, 164.3, 154.9, 138.0, 135.5, 132.6, 126.2, 121.2, 112.1, 104.8, 97.5, 83.3, 53.4, 45.8, 12.2. HRMS (EI): calcd for C_18_H_17_NO_2_: 279.12593, found: 279.12498. **mp**: 97.1–97.8 °C.

**Compound 14**: Compound **11** (184 mg, 0.658 mmol) and Meldrum’s acid (1.0 eq, 95 mg, 0.658 mmol) was dissolved in 99% EtOH (5 mL) to which piperidine (0.02 eq, 1.3 µL 0.0130 mmol) and AcOH (0.02 eq, 0.75 µL, 0.0130 mmol) was added. The solution was stirred at r.t. for 20 min before it was heated to 78 °C and stirred for 3 h. After, the mixture was cooled to r.t. and then to 0 °C and allowed to crystallize overnight. The product was filtered and collected as a yellow-green solid in 48% yield. ^1^H NMR (400 MHz, CDCl_3_) δ ppm 12.30 (s, 1 H), 8.67 (s, 1 H), 7.46 (t, *J* = 8.52 Hz, 3 H), 7.13 (d, *J* = 8.33 Hz, 2 H), 6.72 (dd, *J* = 9.01, 2.45 Hz, 1 H), 6.57 (d, *J* = 2.25 Hz, 1 H), 4.67 (s, 2 H), 3.62 (q, *J* = 7.15 Hz, 2 H), 3.08 (s, 1 H), 1.32 (t, *J* = 7.10 Hz, 3 H). ^13^C NMR (100 MHz, CDCl_3_) δ ppm 165.3, 164.1, 157.8, 154.3, 150.4, 136.8, 132.8, 132.0, 126.1, 121.7, 111.3, 109.2, 106.8, 97.7, 83.0, 53.9, 46.4, 12.1. HRMS (ESI): calcd for C_21_H_17_NO_4_Na ([MNa]^+^): 370.1055, found: 370.1065. **mp**: 207.3–208.8 °C.

**Compound 10 and 15**: POCl_3_ (3.0 eq, 40.4 µL, 432 µmol) was added to a solution of compound **5** (50 mg, 144 µmol) dissolved in DCE (1.4 mL) after which the solution was heated to 84 °C and stirred for 4 h to form the acyl chloride. The solvent was removed by rotary evaporation and the residue was dissolved in acetonitrile (1.4 mL). A separate solution containing **5** or **4** (to make **10** or **15** respectively) (1.2 eq., 49 mg, 173 µmol) and DIPEA (2.0 eq, 50 µL, 288 µmol) in acetonitrile (1.4 mL) was then added to the solution of the acyl chloride and the combined reaction mixture was stirred at room temperature overnight. The solvent was removed by rotary evaporation and the crude was purified by flash chromatography using a gradient elution system of 0%–50% EtOAc in hexanes elution system to afford the product.

**Compound 10:** yellow solid in 28% yield. ^1^H NMR (600 MHz, CDCl_3_) δ ppm 11.00 (s, 1H), 8.77 (s, 1H), 7.83 (d, *J* = 1.9 Hz, 2H), 7.51–7.41 (m, 3H), 7.23 (t, *J* = 1.8 Hz, 1H), 7.14 (d, *J* = 8.3 Hz, 2H), 6.67 (dd, *J* = 8.9, 2.5 Hz, 1H), 6.56 (d, *J* = 2.3 Hz, 1H), 6.48 (q, *J* = 1.7 Hz, 2H), 4.64 (s, 2H), 3.59 (q, *J* = 7.1 Hz, 2H), 3.07 (s, 1H), 2.17 (d, *J* = 1.8 Hz, 6H), 1.30 (t, *J* = 7.1 Hz, 3H). ^13^C NMR (150 MHz, CDCl_3_) δ 170.2, 169.1, 162.9, 161.2, 157.7, 153.6, 149.0, 146.0, 139.4, 137.5, 132.9, 132.7, 131.6, 127.7, 126.3, 121.6, 118.3, 116.5, 110.8, 109.4, 97.7, 83.3, 77.7, 54.0, 46.4, 12.3, 11.3. HRMS (ESI): C_37_H_28_N_4_O_7_ calcd for: 663.1856, found: 663.1855 ([MNa]^+^). **mp**: decomp. at 330.0–331.2 °C.

**Compound 15**: yellow solid in 37% yield. ^1^H NMR (600 MHz, DMSO) δ ppm 10.93 (s, 1H), 8.74 (s, 1H), 7.79 (d, *J* = 1.8 Hz, 2H), 7.74 (d, *J* = 9.1 Hz, 1H), 7.46 (d, *J* = 8.3 Hz, 2H), 7.23 (d, *J* = 8.4 Hz, 2H), 7.21 (s, 4H), 7.11 (t, *J* = 1.8 Hz, 1H), 6.86 (d, *J* = 11.2 Hz, 1H), 6.70 (s, 1H), 4.78 (s, 2H), 4.16 (s, 1H), 3.64 (q, *J* = 7.0 Hz, 2H), 1.19 (t, *J* = 7.1 Hz, 3H). ^13^C NMR (150 MHz, DMSO) δ 169.7, 162.0, 161.0, 157.1, 153.4, 148.3, 138.8, 134.8, 132.4, 132.0, 131.9, 127.7, 126.8, 126.0, 120.4, 120.2, 117.1, 110.9, 109.8, 108.4, 96.8, 83.3, 80.8, 52.8, 45.7, 29.8, 12.2. HRMS (ESI): calcd for C_35_H_24_N_4_O_7_Na ([MNa]^+^): 635.1543, found: 635.1537. **mp**: decomp. at 184.8–184.9 °C.

## 3. Results and Discussion

### 3.1. Initial Design

**Scheme 2:**
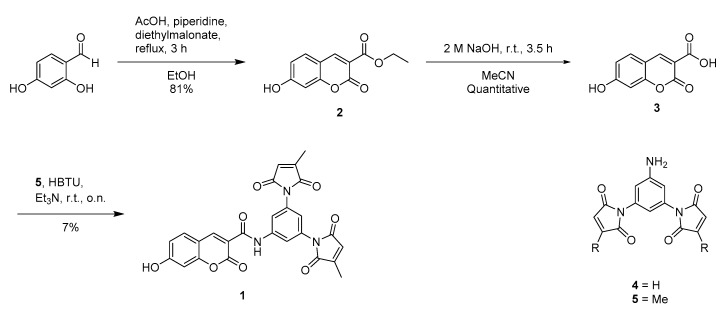
Synthetic route for the preparation of fluorogen **1**.

We initially envisioned attaching a dimaleimide quencher moiety at the C_3_ position of a coumarin fluorophore through an amide bond, while appending a hexyne tail at the C_7_ position through an ether linkage. Thus, we first synthesized **1** (Scheme 2) to confirm that the coumarin FlARe probe without the alkyne tail could be quenched by the dimaleimide quencher. Fluorogen **1** also allowed us to subsequently determine the photophysical effects of adding the terminal alkyne tail.

First the coumarin core was formed via a Knoevenagel condensation between 4-formylresorcinol and diethylmalonate, providing ester **2** in good yield (Scheme 2). Carboxylic acid **3** was then obtained in excellent yield after hydrolysis of **2.** We originally attempted to couple acid **3** with aniline **4**; however, the instability of the unsubstituted maleimide groups of aniline **4** made this synthetic strategy difficult and impractical. Therefore, we switched to aniline **5**, whose methyl-substituted maleimide groups are more stable. Aniline **5** was coupled to carboxylic acid **3** using HBTU to obtain the FlARe probe **1** [29]. The low yield of the final coupling reaction is probably due to the low nucleophilicity of substituted aniline **5**.

**Figure 1:**
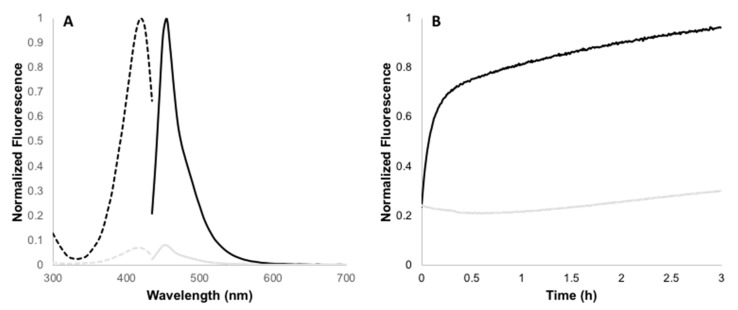
(**A**) Excitation (dotted line) and emission (solid line) spectra of **1** before (grey line) and after reacting with MBP-dC10* (black line). (**B**) Kinetic time course for the fluorogenic reaction between 10 µM of **1** with 10 µM of MBP-dC10* (black), relative to the stability of **1** in the absence of MBP-dC10* (grey). Rate constant *k*_2_ = 245 ± 8 M^−1^s^−1^.

**Scheme 3:**
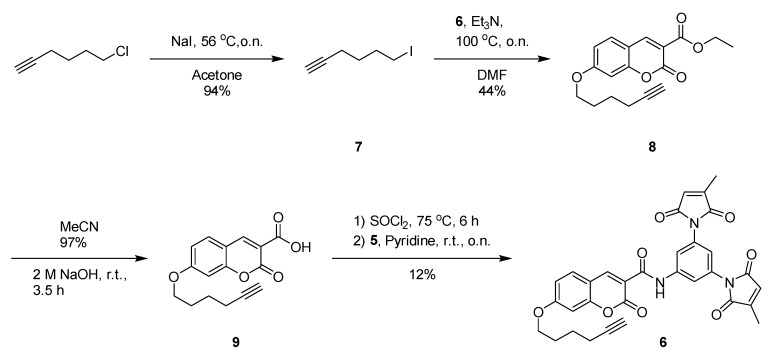
Synthetic route for the preparation of alkyne **6**.

Probe **1** was then reacted with an equimolar concentration of a representative target protein–namely, maltose binding protein genetically fused to the dC10* target peptide (MBP-dC10*), in order to validate the activation of the probe by FlARe (Figure 1) [22]. The reaction of **1** with MBP-dC10* was observed to be fluorogenic with a fluorescence enhancement of 12.7. This is considerably lower than the FE manifested by a previous FlARe probe comprised of a 7-(diethyl)aminocoumarin instead of the 7-hydroxylcoumarin [19]. Notably, the overall fluorescence intensities of both the activated and quenched form of **1** are over 10-fold higher than those of the previously reported **YC5** [19]. Regarding reaction kinetics, the majority of 10 µM **1** reacts with 10 µM dC10* tag within 40 minutes, while the reaction was essentially complete after 2 hours (Figure 1B). From the time course of this reaction, the second order rate constant was calculated as *k*_2_ = 245 ± 8 M^−1^s^−1^. This rate constant is similar to that measured for our previous 7-(diethyl)aminocoumarin FlARe probe [19], suggesting that the change of substituent at the C_7_ position on the coumarin did not influence reactivity. Encouraged by these results, we proceeded to append the terminal hexyne tail through an ether linkage at the C_7_ hydroxyl group, in the synthesis of compound **6** (Scheme 3).

In this approach, we attached the hexyne group before attaching the dimaleimide quenching intermediate **5** (Scheme 3). Thus, **7** was first synthesized from 6-chlorohexyne before using **2** to displace the iodide in **7**, obtaining **8** in decent yield. As performed in the synthesis of **1**, ester **8** was then hydrolyzed to provide **9** in excellent yield, which was then converted to the acid chloride using SOCl_2_. The acid chloride was then reacted with aniline **5** in pyridine to attach the quenching dimaleimide moiety and form **6**. This coupling of aniline **5** also gave a low yield, although slightly better than the HBTU-mediated reaction (Scheme 2).

As before, this novel FlARe linker was incubated with an equimolar concentration of MBP-dC10* (10 µM). However, no fluorescence increase was observed, even after 24 h of incubation (data not shown). Since **6** only differs from **1** by the presence of the terminal hexyne tail, this functional group was suspected of disrupting the coumarin’s intrinsic fluorescence. Density Functional Theory (DFT) calculations of **6** showed that neither the LUMO nor the HOMO of the alkyne group were of appropriate energy to quench coumarin fluorescence through PeT (Figure S1). Thus, it is unlikely that the coumarin fluorescence of **6** was being quenched by this specific mechanism, as opposed to what has been proposed to be the case for other alkyne-functionalized coumarins [30].

Rather, different studies have shown that the coumarin fluorophore’s emission is very sensitive to the functional group’s rigidity at the C_7_ position [31]. Indeed, this sensitivity is exploited in enzyme activity probes in which a carbohydrate linked to umbelliferone at the C_7_ position is enzymatically cleaved to release a highly fluorescent 7-hydroxylcoumarin fluorophore [32,33]. Therefore, in the case of **6,** the terminal hexyne tail at the C_7_ position offers a means of rotational relaxation that could severely diminish the probe’s fluorescence. It is striking to note that the 7-propargyl coumarin probe developed by Liu, bearing a slightly shorter alkyl chain is indeed fluorescent [15]. Thus, extending the alkyl chain beyond three carbons on 7-alkoxycoumarins apparently has a detrimental effect on fluorescence.

**Figure 2:**
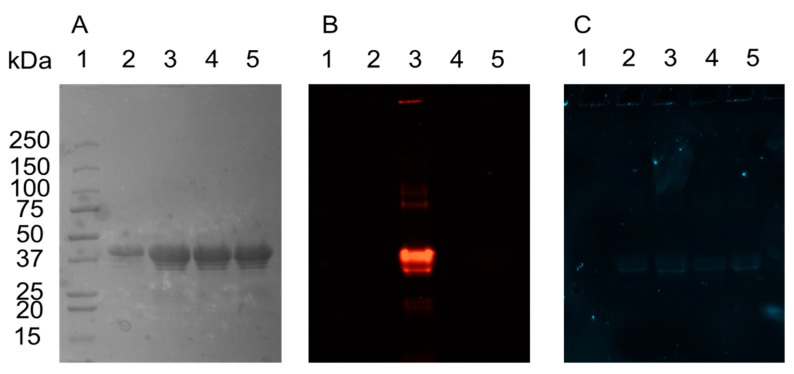
SDS PAGE gel of protein conjugates obtained after first reacting **6** with MBP-dC10* by the FlARe reaction followed by the conjugation of rhodamine B azide (RhB-N_3_) to MBP-dC10* through **6** using CuAAC. (**A**) Coomassie stained gel showing MBP-dC10* bands. (**B**) Unstained gel excited with Green Epi Illumination and imaged using a 605/50 filter. (**C**) Unstained gel excited with Blue Epi Illumination and imaged with a 530/28 filter. Lanes: (1) Pre-stained protein standards (2) MBP-dC10* (3) MBP-dC10* reacted with **6** and then conjugated to RhB-N_3_ (4) MBP-dC10* reacted with **6** (5) MBP-dC10* incubated with RhB-N_3_ under CuAAC conditions.

Despite the lack of fluorogenic activation by FlARe, we tested whether **6** could successfully introduce an alkyne group site-specifically to a target protein, albeit in a non-fluorogenic manner. Specifically, we tested the utility of **6** to couple RhB-N_3_ to MBP-dC10* through a CuAAC conjugating reaction. We first reacted MBP-dC10* with **6** for 24 h to ensure complete labelling. Afterwards, we performed the CuAAC with RhB-N_3_ and evaluated the bioconjugation by fluorescent SDS-PAGE (Figure 2). On the gel, we observed a protein band that is red fluorescent but not blue fluorescent (Figure 2B, lane 3). This was indicative of formation of the MBP-dC10*-RhB-N_3_ conjugate using compound **6** as the linker. The lack of any blue fluorescence is consistent with the non-fluorogenic nature of **6** that was previously observed (Figure 2C). The protein bands in lane 5 of Figure 2 did not fluoresce at all, confirming that without first introducing the alkyne handle using **6**, RhB-N_3_ could not be conjugated to MBP-dC10*. In conclusion, although compound **6** was not observed to be fluorogenic as intended, it still introduced an alkyne group into MBP-dC10*, to which RhB-N_3_ could then be conjugated through cycloaddition.

### 3.2. Improved Design

**Scheme 4:**
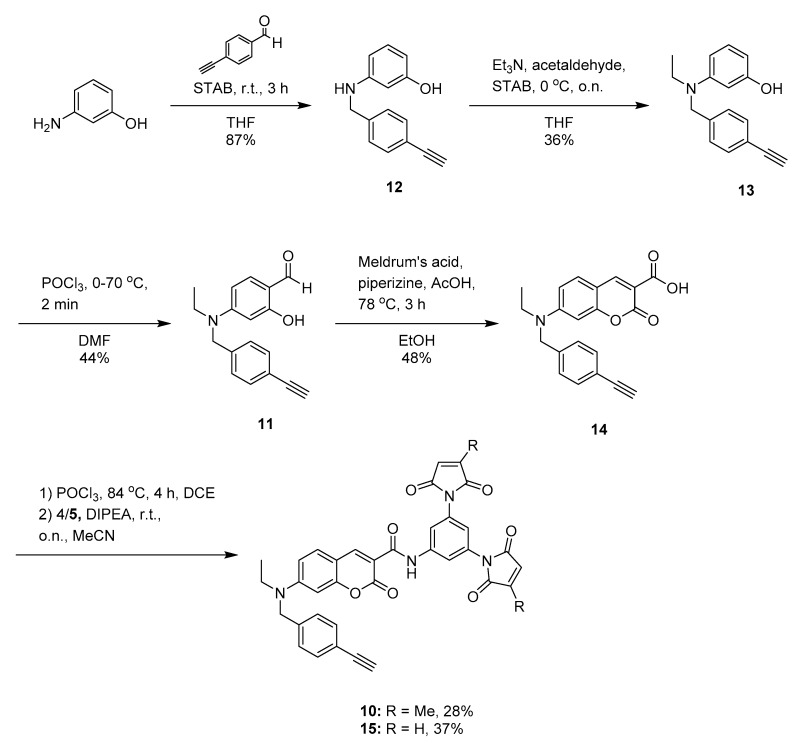
Synthetic route for the preparation of fluorogenic alkynes **10** and **15**.

As previously discussed, the non-fluorogenic nature of **6** made it difficult to ascertain how rapidly and efficiently the alkyne group was introduced. To address this issue, we designed a second fluorogenic bioconjugating linker where the alkyne group was attached to the coumarin core at the C_7_ position as a 4-ethynylbenzylamine substituent. We hypothesized that this functional group would present less rotational flexibility and maintain the coumarin’s intrinsic fluorescence. These modifications are comprised in the design of the fluorogenic linker **10** shown in Scheme 4.

Prior to the formation of **10**, the salicylaldehyde **11** was prepared (Scheme 4). First, the terminal alkyne was attached by reacting 3-aminophenol with *p*-ethynylbenzaldehyde in a reductive amination using sodium triacetoxyborohydride (STAB). This afforded the secondary amine **12** in good yield, which was then capped with acetaldehyde through a second reductive amination using STAB to yield the tertiary amine **13** in moderate yield. A Vilsmeier–Haack reaction was performed to obtain the functionalized salicylaldehyde **11** in moderate yield, which was then followed by a Knoevenagel condensation with Meldrum’s acid to obtain coumarin **14**. Finally, the di(methylmaleimide) aniline intermediate **5** was attached to **14** by first converting the latter to its acid chloride with POCl_3_ and then forming the amide bond to afford **10** in good yield.

**Figure 3:**
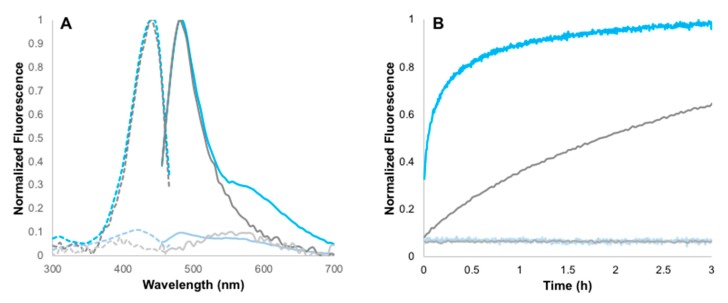
(**A**) Excitation (dotted line) and emission (solid line) spectra of 10 µM **10** (grey lines) and **15** (blue lines) before (light) and after (dark) reacting with 10 µM MBP-dC10*. (**B**) Kinetic time course for the fluorogenic reaction between 10 µM of **10** (grey lines) and **15** (blue lines) with 10 µM of MBP-dC10* (dark lines) (black), relative to the stability of **10** and **15** in the absence of MBP-dC10* (light lines).

Reacting equimolar amounts of **10** with MBP-dC10* at 10 µM resulted in a 37-fold enhancement in fluorescence after complete reaction with the tag (Figure 3A and Figure S2A). Not only did this indicate successful fluorescence activation by FlARe, it is also a significant improvement from the FE observed with probe **1.** However, nearly 10 h were required for the complete reaction of **10** with MBP-dC10* at 10 µM, providing a rate constant of *k*_2_ = 8.4 ± 0.7 M^−1^s^−1^, which is over an order of magnitude lower than the rate constant measured for **1** (Figure S2B). This suggests that the modified alkyne tail of **6** may hinder the labelling reaction. On a practical level, this means that making micromolar concentrations of a protein conjugate would require at least two days to complete, calling for a quicker alternative. As mentioned before, unsubstituted maleimides react more quickly than methylmaleimides [29]. Thus, the di(methylmaleimide) aniline moiety was replaced with dimaleimide aniline in the design of probe **15** (Scheme 4).

Using the same synthetic strategy outlined in Scheme 4, aniline **4** was coupled to **14** through the acyl chloride afforded by POCl_3_ to obtain **15** in decent yield. Upon reacting with equimolar amounts of MBP-dC10* at 10 µM, the fluorescence intensity of **15** was found to increase 10-fold. The final MBP-dC10*-**15** adduct displayed a quantum yield of Φ = 0.093, which is 57 times higher than the quantum yield of the unreacted form of **15** (Figure S3). This FE was three-fold lower compared to **10** but still comparable to the FE observed with **1** (Figure 3A). The rate constant of the reaction between the target peptide and **15** was calculated to be *k*_2_ = 180 ± 10 M^−1^s^−1^, with completion at the 10-µM concentrations achieved after only 30 minutes (Figure 3B). Thus, switching to the unsubstituted maleimide accelerated the labelling reaction by over an order of magnitude, making it comparable to the reaction between **1** and MBP-dC10*.

**Figure 4:**
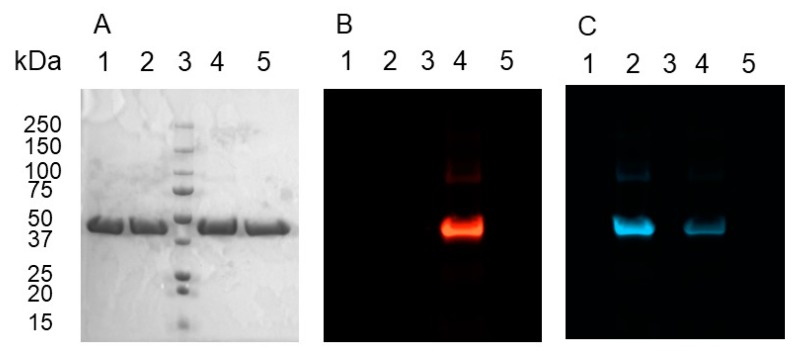
SDS PAGE gel of protein conjugates obtained after first reacting 10 µM **15** with 10 µM MBP-dC10*, followed by the conjugation of rhodamine B azide (RhB-N_3_) to MBP-dC10* through **15** using CuAAC. (**A**) Coomassie stained gel showing MBP-dC10* bands. (**B**) Unstained gel imaged using a 605/50 filter. (**C**) Unstained gel excited with Blue Epi Illumination and imaged with a 530/28 filter. Lanes: (1) MBP-dC10* (2) MBP-dC10* reacted with **15** (3) pre-stained protein molecular weight markers (4) MBP-dC10* reacted with **15** and then conjugated to RhB-N_3_ (5) MBP-dC10* incubated with RhB-N_3_ under CuAAC conditions.

Furthermore, the total time required to prepare a protein conjugate was reduced to a one-day process. Encouraged by both the fluorogenic nature of **15** and its rapid labelling reaction, the utility of **15** for bioconjugation was assessed in a manner similar to **6**. Thus, 10 µM of **15** was incubated with 10 µM MBP-dC10* for 1 h, after which RhB-N_3_ was then conjugated to MBP-dC10* by CuAAC through the alkyne handle introduced by **15**. SDS-PAGE analysis showed a protein band that was both red *and* blue fluorescent (Figure 4B,C, lane 4), indicating that MBP-dC10* was conjugated to the red fluorescent rhodamine B through the blue fluorescent **15** coumarin linker. Interestingly, the blue fluorescence of this protein band is considerably dimmer than MBP-dC10* labelled only with **15** (Figure 4C, lane 2). We suspect this is because of a FRET interaction between the coumarin fluorophore of **15** and the rhodamine B group of the model ‘cargo’. This FRET interaction has been identified previously in the literature, and a comparison of the coumarin emission spectrum of the adduct of **15** and the excitation spectrum of rhodamine B (see Figure S4) reveals the significant spectral overlap required for FRET [20,34,35]. Furthermore, molecular modelling of the triazole adduct formed after CuAAC coupling suggests the fluorophores would be separated by less than 30 Å, allowing for efficient resonance energy transfer. The protein band in lane 5 is neither red nor blue fluorescent, confirming that the linker **15** is required to successfully conjugate RhB-N_3_ to MBP-dC10* (Figure 4B, lane 5). Therefore, **15** successfully introduced an exposed alkyne group in a site-specific and fluorogenic manner, which allowed for the attachment of the azide functionalized cargo, RhB-N_3_.

**Figure 5:**
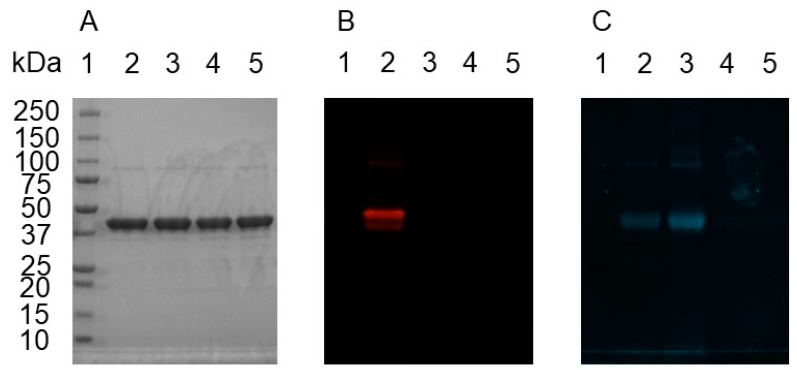
SDS PAGE analysis of protein conjugates obtained after first reacting **15** with rhodamine B azide (RhB-N_3_) in the CuAAC reaction, followed by conjugation to MBP-dC10* by the FlARe reaction. (**A**) Coomassie stained gel showing MBP-dC10* bands. (**B**) Unstained gel excited with Green Epi Illumination and imaged using a 605/50 filter. (**C**) Unstained gel excited with Blue Epi Illumination and imaged with a 530/28 filter. Lanes: (1) Pre-stained protein standards (2) MBP-dC10* reacted with the **15**-RhB-N_3_ product of the CuAAC. (3) MBP-dC10* reacted with **15** subjected to a mock CuAAC without RhB-N_3_. (4) MBP-dC10* incubated with RhB-N_3_ under CuAAC conditions. (5) MBP-dC10*.

In addition to its rapid kinetics, **15** was also noted to be resistant to hydrolysis over the course of 3 h under the assay conditions. This prompted us to perform the CuAAC between **15** and RhB-N_3_ *prior* to conjugation of the triazole product to MBP-dC10*, as we hypothesized that the maleimides would remain intact after the CuAAC. Thus, after performing the CuAAC between **15** and RhB-N_3_, we added MBP-dC10* in equimolar concentrations to **15** and allowed the **15**-RhB triazole product to react with the dC10* tag for 1 h. As before, the bioconjugation was analysed by SDS-PAGE (Figure 5). The presence of a red and blue fluorescent protein band (Figure 5B,C, lane 2) indicates the successful formation of the MBP-dC10*-rhodamine conjugate. Like the previous experiment, in the lane representing the conjugation of the RhB-N_3_ cargo to MBP-dC10* *after* labelling the protein with **15**, the blue fluorescence of this band is dimmer than that of MBP-dC10* labelled only with **15**, and no red fluorescence is observed from the band in lane 4. This reflects both the FRET interaction occurring between the fluorophores and the importance of **15** for successful bioconjugation.

However, it is also noteworthy that the intensity of the fluorescence of *all* the protein bands shown in Figure 5 appears to be lower than those from the previous experiment (shown in Figure 4). The similarity of the density of the Coomassie stained protein bands between the gels (Figure 4A vs. Figure 5A) suggests this is not due to a difference in gel loading. We hypothesize that the labelling reaction of the **15**-RhB triazole adduct with MBP-dC10* (shown in Figure 5) was less efficient than the reaction of **15** with MBP-dC10* (shown in Figure 4), leading to a lower proportion of protein being labelled after 1 hour of incubation, and a less intense signal being observed. It is conceivable that the steric bulk of the adduct pre-formed between rhodamine B and fluorogen **15** decreases the efficiency of the subsequent reaction with MBP-dC10*, slowing the labelling reaction in a manner similar to what was observed on comparing **1** and **10**. Therefore, although we have demonstrated that it is possible to perform the FlARe protein-labelling reaction either before or after the CuAAC reaction, it appears to be more efficient to label the protein first and then attach the cargo to the labelled protein through the alkyne group.

## 4. Conclusions

In summary, we have developed a new bioconjugation method that first introduces an alkyne group onto a target protein in a site-specific, rapid and fluorogenic manner. This allows for the subsequent site-specific attachment of a broad array of azide-functionalized cargo. Our initially designed bifunctionalized conjugating agent (**6**) proved to be non-fluorescent, presumably due to its long, flexible hexyne tail. Despite being non-fluorogenic, **6** was still capable of introducing an alkyne group to the test protein MBP-dC10*, onto which the model cargo RhB-N_3_ could then be attached. In our second design, the long alkyne tail of **6** was replaced with a shorter and more rigid alkynyl amine substituent, while the di(methylmaleimide) aniline was replaced with dimaleimide aniline, resulting in the heterobifunctional fluorogenic linker **15**. These modifications resulted in a linker whose fluorescence increased 10-fold upon reacting with the dC10* target peptide (Φ = 0.093), allowing the introduction of the exposed alkyne group to be monitored in situ by fluorescence increase. Subsequently, model cargo RhB-N_3_ could be attached to MBP-dC10* through the site-specifically installed alkyne handle presented by **15**. Furthermore, we demonstrated that azide-functionalized cargo can also be incorporated into **15** by CuAAC *prior* to reacting with the target protein. Overall, our method allows the site-specific introduction of an exposed alkyne group into a protein of interest genetically fused to a dC10* tag, representing an approach that is easily amenable for the synthesis of new, therapeutically important protein-cargo conjugates. Additionally, the fluorogenic nature of the bioconjugating linker allows the initial protein modification step to be easily monitored in situ by fluorescence. Therefore, our linker provides a facile, modular and adaptable method for the in vitro synthesis of new protein-cargo conjugates in the future.

## Supplementary Materials

The following are available online at https://www.mdpi.com/2218-273X/10/3/369/s1, Figure S1, Figure S2, Figure S3, Figure S4, Figure S5, synthetic protocols for RhB-N_3_, compound **4** and its intermediates, ^1^H-NMR and ^13^C-NMR spectra.

## References

[1] Pasut G., Veronese F.M. (2009). PEG conjugates in clinical development or use as anticancer agents: An overview. Adv. Drug Deliv. Rev..

[2] Sievers E.L., Senter P.D. (2013). Antibody-Drug Conjugates in Cancer Therapy. Annu. Rev. Med..

[3] Canalle L.A., Löwik D.W.P.M., Van Hest J.C.M. (2010). Polypeptide-polymer bioconjugates. Chem. Soc. Rev..

[4] Deonarain M.P., Yahioglu G., Stamati I., Marklew J. (2015). Emerging formats for next-generation antibody drug conjugates. Expert Opin. Drug Discov..

[5] Chudasama V., Maruani A., Caddick S. (2016). Recent advances in the construction of antibody-drug conjugates. Nat. Chem..

[6] Veronese F.M. (2001). Peptide and protein PEGylation: a review of problems and solutions. Biomaterials.

[7] Yamada K., Ito Y. (2019). Recent Chemical Approaches for Site-Specific Conjugation of Native Antibodies: Technologies toward Next-Generation Antibody–Drug Conjugates. ChemBioChem.

[8] Kim J.S., Sirois A.R., Vazquez Cegla A.J., Jumai’An E., Murata N., Buck M.E., Moore S.J. (2019). Protein-Polymer Conjugates Synthesized Using Water-Soluble Azlactone-Functionalized Polymers Enable Receptor-Specific Cellular Uptake toward Targeted Drug Delivery. Bioconjug. Chem..

[9] Klok H.A. (2009). Peptide/protein-synthetic polymer conjugates: Quo vadis. Macromolecules.

[10] Duncan R. (2003). The dawning era of polymer therapeutics. Nat. Rev. Drug Discov..

[11] Pelegri-Oday E.M., Lin E.W., Maynard H.D. (2014). Therapeutic protein-polymer conjugates: Advancing beyond pegylation. J. Am. Chem. Soc..

[12] Ducry L., Stump B. (2010). Antibody-drug conjugates: Linking cytotoxic payloads to monoclonal antibodies. Bioconjug. Chem..

[13] Agarwal P., Bertozzi C.R. (2015). Site-specific antibody-drug conjugates: The nexus of bioorthogonal chemistry, protein engineering, and drug development. Bioconjug. Chem..

[14] Liu G., Hu J., Liu S. (2018). Emerging Applications of Fluorogenic and Non-fluorogenic Bifunctional Linkers. Chemistry..

[15] Liu G., Shi G., Sheng H., Jiang Y., Liang H., Liu S. (2017). Doubly Caged Linker for AND-Type Fluorogenic Construction of Protein/Antibody Bioconjugates and In Situ Quantification. Angew. Chem. Int. Ed. Engl..

[16] Robin M.P., Wilson P., Mabire A.B., Kiviaho J.K., Raymond J.E., Haddleton D.M., O’Reilly R.K. (2013). Conjugation-induced fluorescent labeling of proteins and polymers using dithiomaleimides. J. Am. Chem. Soc..

[17] Dirks A.T.J., Cornelissen J.J.L.M., Nolte R.J.M. (2009). Monitoring Protein- Polymer Conjugation by a Fluorogenic Cu (I)-Catalyzed Azide- Alkyne 1, 3-Dipolar Cycloaddition. Bioconjug. Chem..

[18] Chen Y., Tsao K., Acton S.L., Keillor J.W. (2018). A Green BODIPY-Based, Super-Fluorogenic, Protein-Specific Labelling Agent. Angew. Chemie Int. Ed..

[19] Chen Y., Clouthier C.M., Tsao K., Strmiskova M., Lachance H., Keillor J.W. (2014). Coumarin-based fluorogenic probes for no-wash protein labeling. Angew. Chem. Int. Ed. Engl..

[20] Chen Y., Tsao K., Keillor J.W. (2014). Fluorogenic protein labelling: a review of photophysical quench mechanisms and principles of fluorogen design. Can. J. Chem..

[21] Guy J., Caron K., Dufresne S., Michnick S.W., Skene W.G., Keillor J.W. (2007). Convergent preparation and photophysical characterization of dimaleimide dansyl fluorogens: elucidation of the maleimide fluorescence quenching mechanism. J. Am. Chem. Soc..

[22] Strmiskova M., Tsao K., Keillor J.W. (2018). Rational design of a highly reactive dicysteine peptide tag for fluorogenic protein labelling. Org. Biomol. Chem..

[23] Guy J., Castonguay R., Campos-Reales Pineda N.B., Jacquier V., Caron K., Michnick S.W., Keillor J.W. (2010). De novo helical peptides as target sequences for a specific, fluorogenic protein labelling strategy. Mol. Biosyst..

[24] Nasheri N., Joyce M., Rouleau Y., Yang P., Yao S., Tyrrell D.L., Pezacki J.P. (2013). Modulation of fatty acid synthase enzyme activity and expression during hepatitis C virus replication. Chem. Biol..

[25] Yang P.Y., Liu K., Ngai M.H., Lear M.J., Wenk M.R., Yao S.Q. (2010). Activity-based proteome profiling of potential cellular targets of orlistat - An FDA-approved drug with anti-tumor activities. J. Am. Chem. Soc..

[26] Abdizadeh T., Kalani M.R., Abnous K., Tayarani-Najaran Z., Khashyarmanesh B.Z., Abdizadeh R., Ghodsi R., Hadizadeh F. (2017). Design, synthesis and biological evaluation of novel coumarin-based benzamides as potent histone deacetylase inhibitors and anticancer agents. Eur. J. Med. Chem..

[27] Jiang X.R., Wang P., Smith C.L., Zhu B.T. (2013). Synthesis of novel estrogen receptor antagonists using metal-catalyzed coupling reactions and characterization of their biological activity. J. Med. Chem..

[28] Takakura H., Sasakura K., Ueno T., Urano Y., Terai T., Hanaoka K., Tsuboi T., Nagano T. (2010). Development of luciferin analogues bearing an amino group and their application as BRET donors. Chem. Asian J..

[29] Chen Y., Tsao K., De Francesco É., Keillor J.W. (2015). Ring Substituent Effects on the Thiol Addition and Hydrolysis Reactions of N-Arylmaleimides. J. Org. Chem..

[30] Jewett J.C., Bertozzi C.R. (2011). Synthesis of a fluorogenic cyclooctyne activated by Cu-free click chemistry. Org. Lett..

[31] Grimm J.B., English B.P., Chen J., Slaughter J.P., Zhang Z., Revyakin A., Patel R., Macklin J.J., Normanno D., Singer R.H. (2015). A general method to improve fluorophores for live-cell and single-molecule microscopy. Nat. Methods.

[32] Kwan D.H., Ernst S., Kötzler M.P., Withers S.G. (2015). Chemoenzymatic Synthesis of a Type 2 Blood Group A Tetrasaccharide and Development of High-throughput Assays Enables a Platform for Screening Blood Group Antigen-cleaving Enzymes. Glycobiology.

[33] Zhang X., Chen F., Petrella A., Chacón-Huete F., Covone J., Tsai T.W., Yu C.C., Forgione P., Kwan D.H. (2019). A High-Throughput Glycosyltransferase Inhibition Assay for Identifying Molecules Targeting Fucosylation in Cancer Cell-Surface Modification. ACS Chem. Biol..

[34] Yuan L., Lin W., Zheng K., Zhu S. (2013). FRET-based small-molecule fluorescent probes: Rational design and bioimaging applications. Acc. Chem. Res..

[35] Zhang X.-F., Zhang T., Shen S.-L., Miao J.-Y., Zhao B.-X. (2015). A ratiometric lysosomal pH probe based on the coumarin–rhodamine FRET system. RSC Adv..

