# Supplementary material for: Site-Specific Fluorogenic Protein Labelling Agent for Bioconjugation
Source: Biomolecules. 2020 Feb 28;10(3):369. doi: 10.3390/biom10030369 (PMC7175205; doi:10.3390/biom10030369)
Supplement: Supplementary file 1 [file biomolecules-10-00369-s001.pdf]

Site-Specific Fluorogenic Protein Labelling Agent for Bioconjugation

Kelvin K. Tsao, Ann C. Lee, Karl É. Racine, Jeffrey W. Keillor*

Department of Chemistry and Biomolecular Sciences, University of Ottawa
10 Marie-Curie, Ottawa, ON K1N 6N5 (Canada)
*Corresponding author: jkeillor@uottawa.ca

Figure S1. 2
Figure S2. 3
Figure S3. 4
Figure S4. 5
Synthesis of RhB-N_3 6
Figure S5. 7
Synthesis of Compound 4 7
References 8
NMR Spectra 9

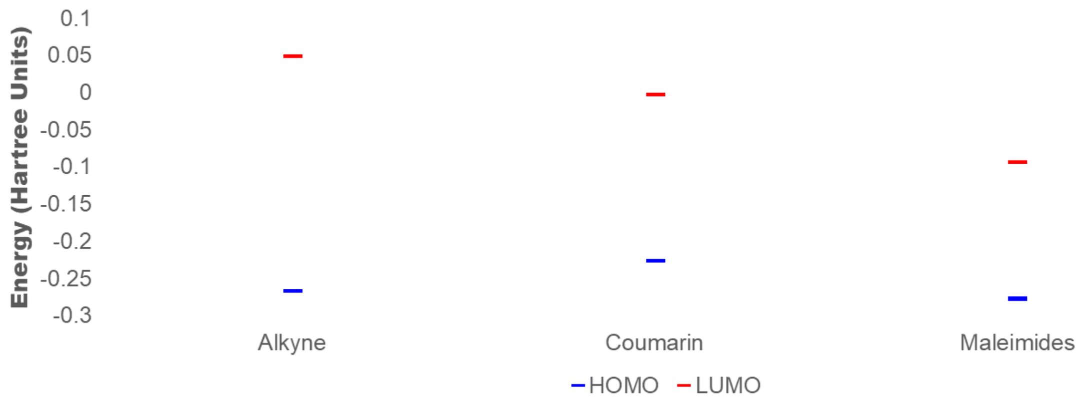

Figure S1.
HOMO and LUMO energies of the alkyne, coumarin and maleimides in 6 and their relative positions. Density functional theory (DFT) was used to optimize the geometry of ground-state structures using the B3LYP functional and 6-31G(d) set with water as solvent. Time-dependent DFT (TD-DFT) calculations were then performed at the B3LYP/6-31G(d) level based on the optimized structure of the ground state. All calculations were performed using Gaussian 09.

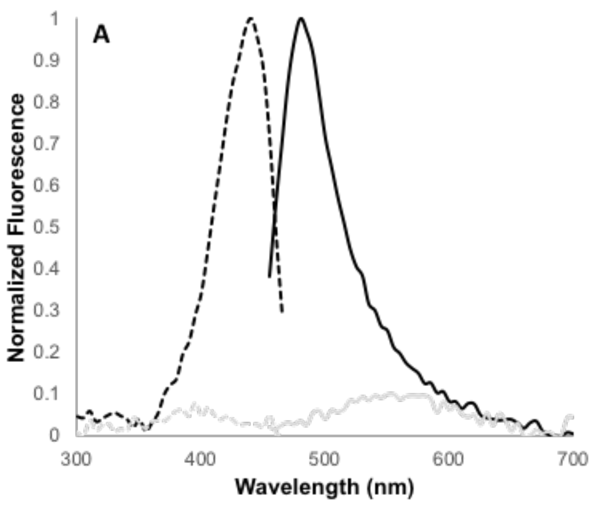

<u>**Figure S2.**</u>
(A) Excitation (dotted line) and emission (solid line) spectra of **10** before (grey line) and after (black line) reacting with MBP-dC10* for 24 h. (B) Kinetic time course for the fluorogenic reaction between 10 μM of **10** with 10 μM of MBP-dC10* (black), relative to the stability of **10** in the absence of MBP-dC10* (grey) k_2 = 8.4 ± 0.7 $M^{-1}s^{-1}$.

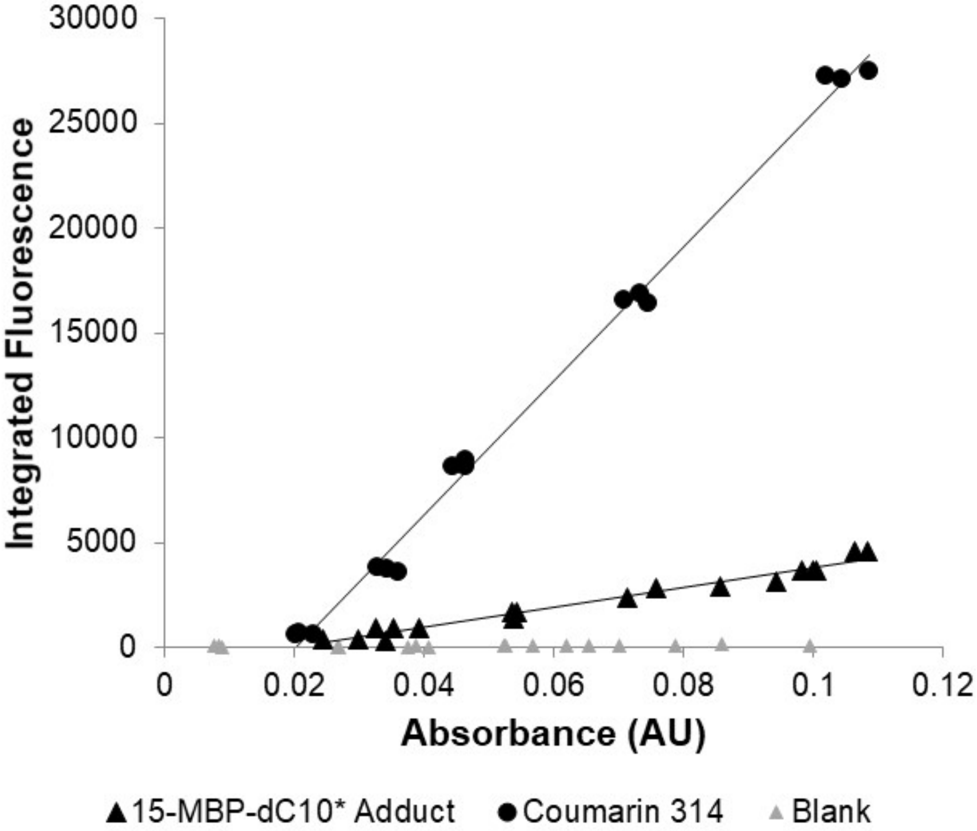

Figure S3.

Integrated fluorescence vs. absorbance of the coumarin 314 standard (black circles, $m_{std} = 32.0 \pm 0.7 \times 10^4$, $R^2 = 0.9946$) and the **15**-MBP-dC10* adduct (black triangles, $m_{sample} = 4.8 \pm 0.2 \times 10^4$, $R^2 = 0.9704$) for determining the quantum yield of activated **15**. The blank (grey triangles, $m_{sample} = 0.08 \times 10^4$, $R^2 = 0.6032$) consisted of unreacted **15** in the absence of MBP-dC10*.

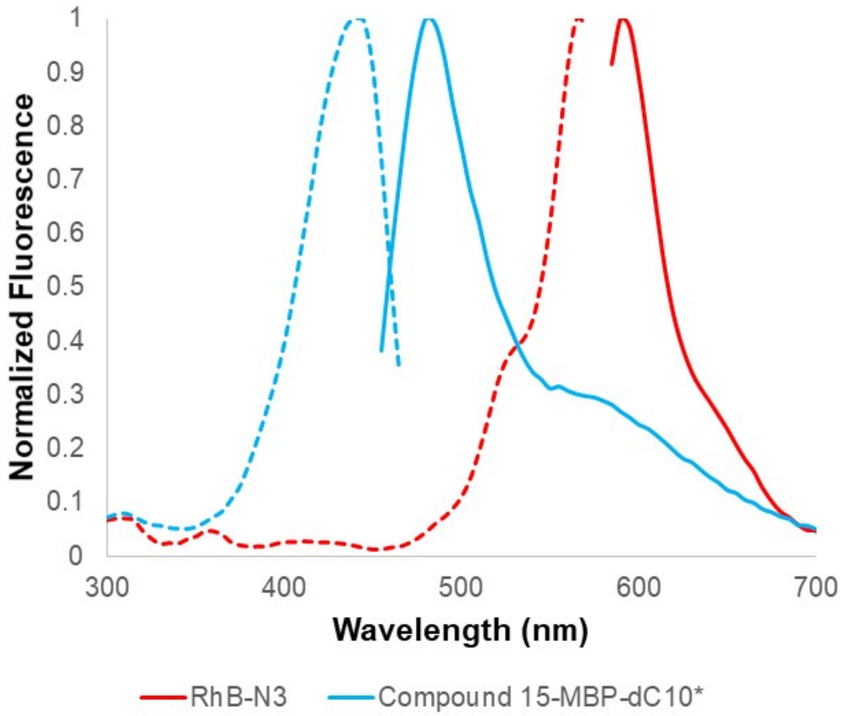

Figure S4.

Normalized excitation (dotted line) and emission (solid line) spectra for RhB-N_3 (red) and the Compound **15**-MBP-dC10* adduct (blue). Note the significant overlap between the coumarin emission spectrum of the adduct (solid blue line) and the excitation spectrum of rhodamine B (dotted red line).

Note: the Compound **15**-MBP-dC10* adduct was formed by reacting 10 μM of compound **15** with 10 μM MBP-dC10* for 4 h, as outlined in the methods, and spectra were recorded thereafter. The spectra recorded for RhB-N_3 were obtained at 25 μM (λ_{ex} = 565 nm, λ_{em} = 588 nm).

Synthesis of RhB-N_3

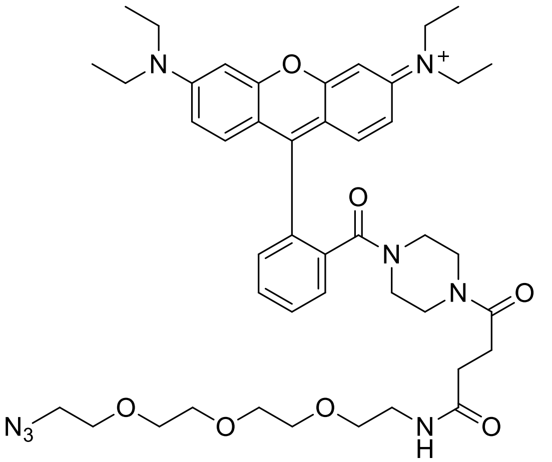

RhB-N_3 was synthesized according to the protocol published by Yang *et al*. [1] Spectral data obtained match reported spectral data.

Figure S5.

Synthesis of **4**.

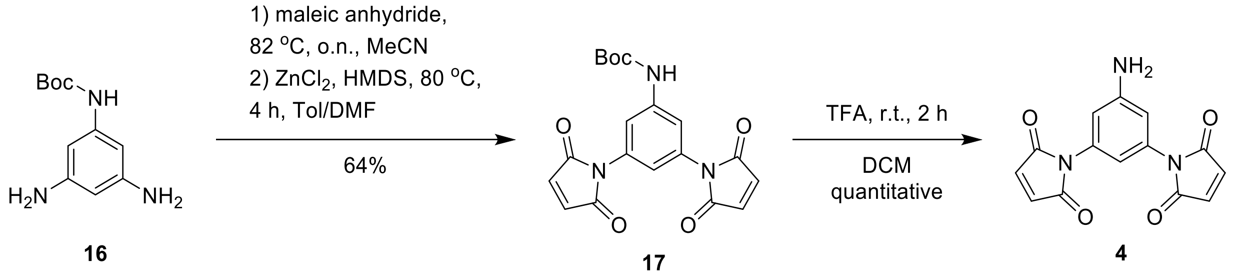

Synthesis of Compound 4

Compound 16: 16 was synthesized using the protocol developed by Chen, *et al.*[2] Spectral data obtained match reported spectral data.

Compound 17: Compound **16** (800 mg, 3.58 mmol) was dissolved in MeCN (35 mL) and to this solution was added maleic anhydride (3.0 eq, 1.05 g, 10.7 mmol). The solution was heated to reflux and stirred overnight. Afterwards, the solvent was removed using the rotary evaporator and the residue was triturated with diethyl ether (3 × 35 mL). The triturated residue was dissolved in DMF (25 mL) and diluted with toluene (100 mL). A solution of HMDS (4.5 eq, 3.36 mL, 16.1 mmol) in toluene (20 mL) was then added dropwise, followed by the addition of $ZnCl_2$ (3.0 eq, 1.46 g, 10.7 mmol). The mixture was heated to 80 °C and stirred for 4 h before being diluted with EtOAc (250 mL). The solution was washed with 0.1 M HCl (3 × 80 mL), sat. $NaHCO_3$ (80 mL) and brine (80 mL) and then dried with $MgSO_4$ and evaporated on the rotary evaporator. The crude was purified by flash chromatography using a 4:6 EtOAc/hexanes elution system. Compound **17** was obtained as a yellow solid in 64% yield. ^{1}H NMR (400 MHz, $CDCl_3$) δ ppm 7.47 (d, *J* = 1.6 Hz, 2H), 7.15 (t, J = 1.8 Hz, 1H), 6.84 (s, 4H), 6.70 (s, 1H), 1.50 (s, 9H). ^{13}C NMR (100 MHz, $CDCl_3$) δ 169.1, 152.4, 139.9, 134.4, 132.5, 117.1, 114.7, 81.4, 28.4. HRMS (ESI): calcd for $C_{19}H_{17}N_3O_6Na$ ($[MNa]^+$): 406.1015, found: 406.1012.

Compound **4**: Compound **17** (877 mg, 2.29 mmol) was dissolved in DCM (10 mL) and TFA (57 eq., 10 mL, 130 mmol) was added dropwise. The solution was stirred at room temperature for 2 h before the liquid reagents and solvents were removed using a rotary evaporator. The crude was triturated with DCM (5 × 50 mL) and compound **4** was obtained as a white solid in quantitative yield. ^{1}H NMR (400 MHz, DMSO) δ ppm 7.16 (s, 4H), 6.66 (d, *J* = 1.5 Hz, 2H), 6.57 (s, 1H). ^{13}C NMR (100 MHz, DMSO) δ 169.7, 147.0, 134.6, 132.4, 113.7, 112.6. HRMS (ESI): calcd for C14H9N3O4Na ($[MNa]^+$): 306.0491, found: 306.0499. **mp**: 175.3 – 177.0 °C.

References

(1) Yang, P. Y.; Liu, K.; Ngai, M. H.; Lear, M. J.; Wenk, M. R.; Yao, S. Q. (2010) Activity-Based Proteome Profiling of Potential Cellular Targets of Orlistat - An FDA-Approved Drug with Anti-Tumor Activities. J. Am. Chem. Soc. 132, 656–666.

(2) Chen, Y.; Clouthier, C. M.; Tsao, K.; Strmiskova, M.; Lachance, H.; Keillor, J. W. (2014) Coumarin-Based Fluorogenic Probes for No-Wash Protein Labeling. Angew. Chem. Int. Ed. Engl. 53, 13785–13788.

NMR Spectra

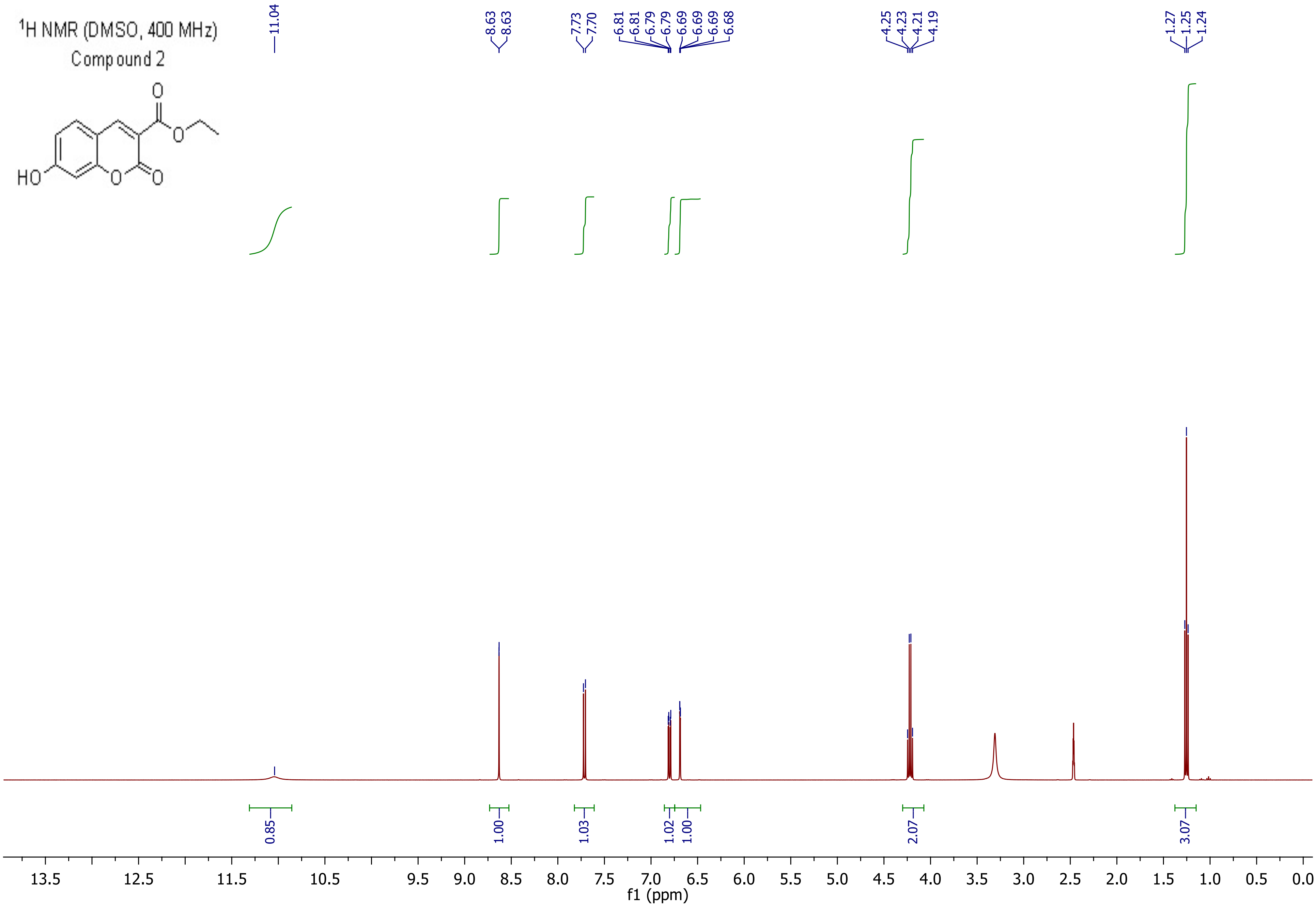

^{1}H NMR (DMSO, 400 MHz)
Compound 2
HO
O
O
O
O
11.04
8.63
8.63
7.73
7.70
6.81
6.81
6.79
6.79
6.69
6.69
6.69
6.68
4.25
4.23
4.21
4.19
1.27
1.25
1.24
0.85
1.00
1.03
1.02
1.00
2.07
3.07
13.5
12.5
11.5
10.5
9.5
9.0
8.5
8.0
7.5
7.0
6.5
6.0
5.5
5.0
4.5
4.0
3.5
3.0
2.5
2.0
1.5
1.0
0.5
0.0
f1 (ppm)

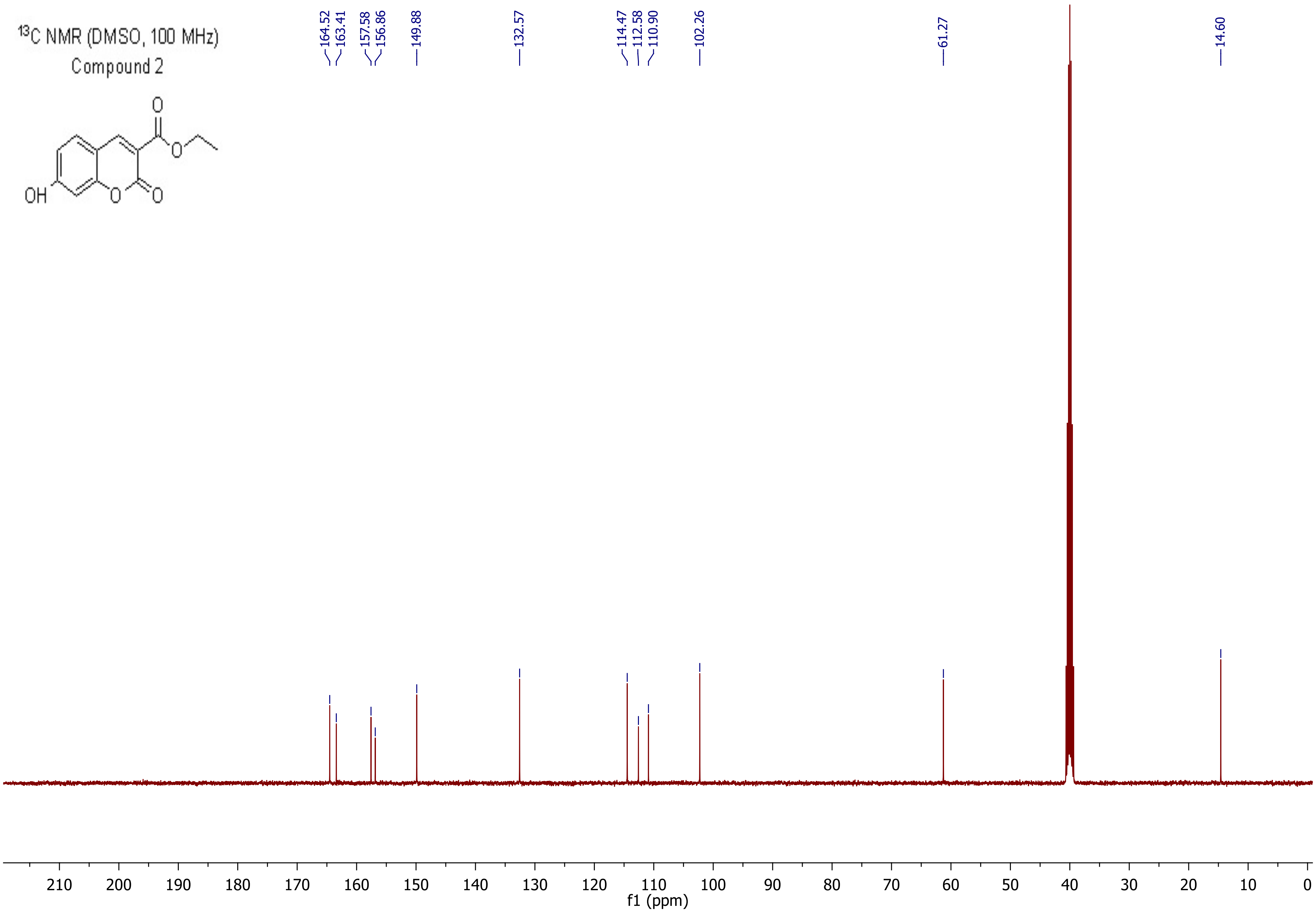

13C NMR (DMSO, 100 MHz)
Compound 2
OH
O
O
O
O
164.52
163.41
157.58
156.86
149.88
132.57
114.47
112.58
110.90
102.26
61.27
14.60
210
200
190
180
170
160
150
140
130
120
110
100
90
80
70
60
50
40
30
20
10
0
f1 (ppm)

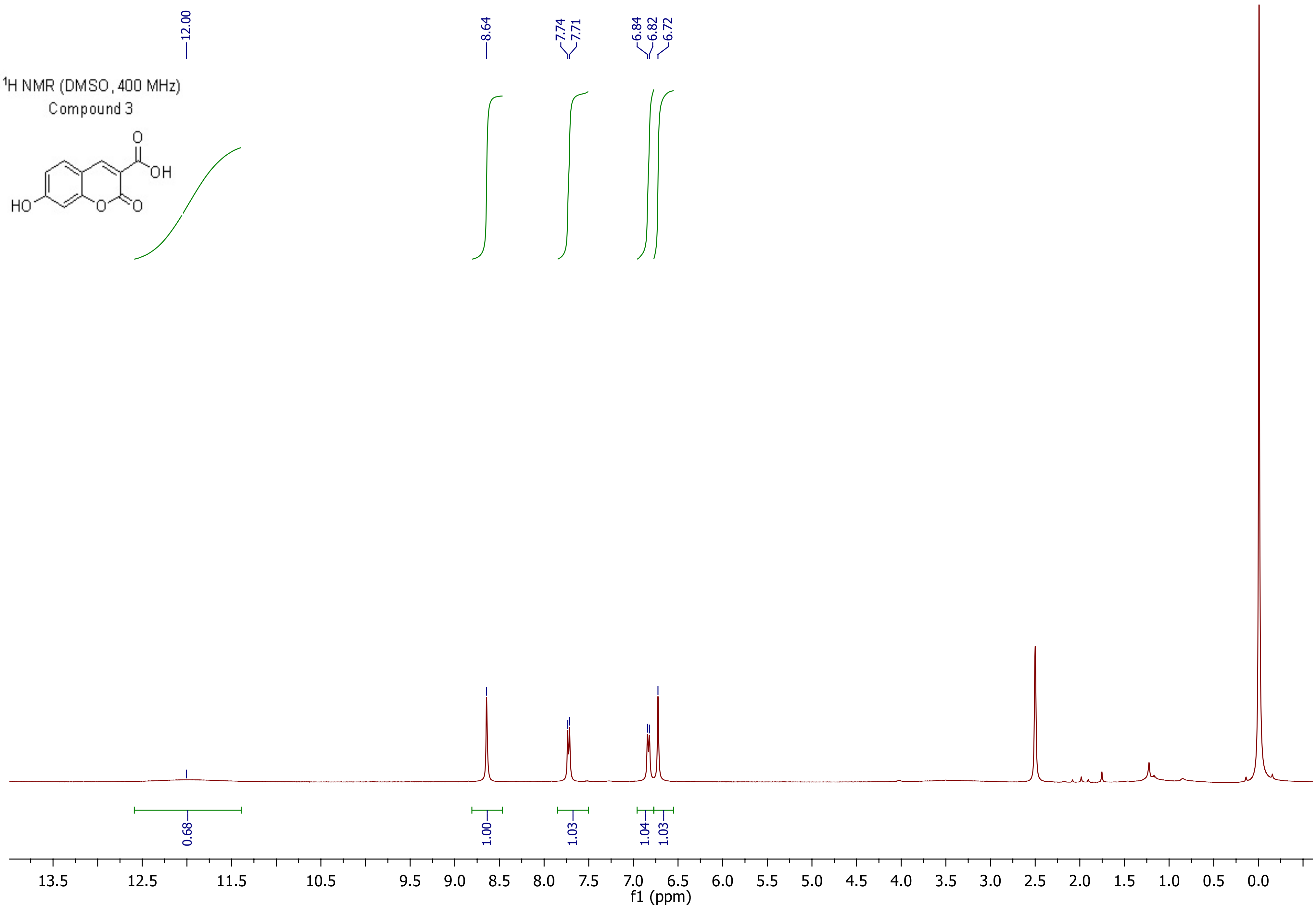

1H NMR (DMSO, 400 MHz)
Compound 3
HO
O
O
O
OH
12.00
8.64
7.74
7.71
6.84
6.82
6.72
0.68
1.00
1.03
1.04
1.03
13.5
12.5
11.5
10.5
9.5
9.0
8.5
8.0
7.5
7.0
6.5
6.0
5.5
5.0
4.5
4.0
3.5
3.0
2.5
2.0
1.5
1.0
0.5
0.0
f1 (ppm)

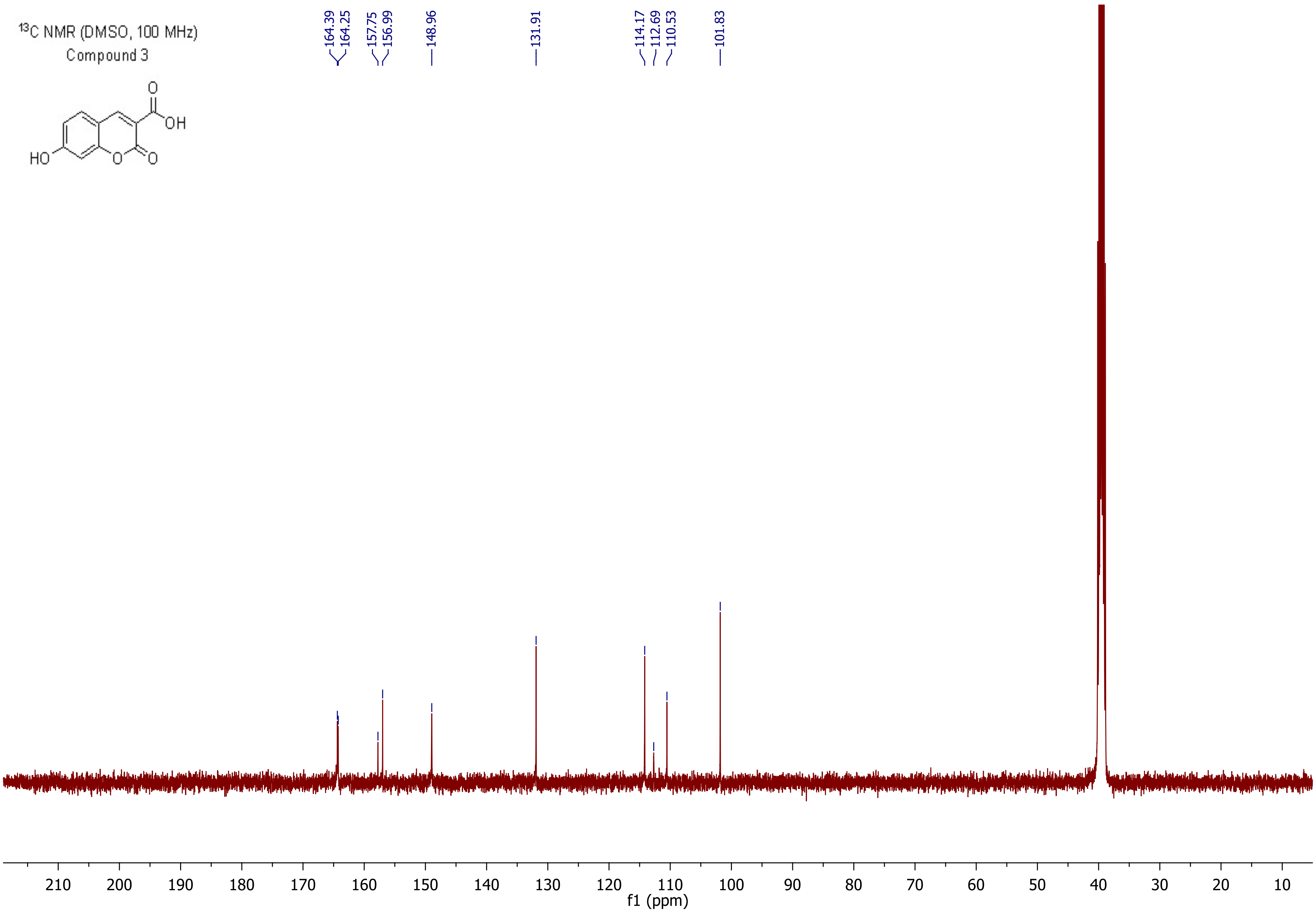

13C NMR (DMSO, 100 MHz)
Compound 3
HO
O
O
O
OH
164.39
164.25
157.75
156.99
148.96
131.91
114.17
112.69
110.53
101.83
210 200 190 180 170 160 150 140 130 120 110 100 90 80 70 60 50 40 30 20 10
f1 (ppm)

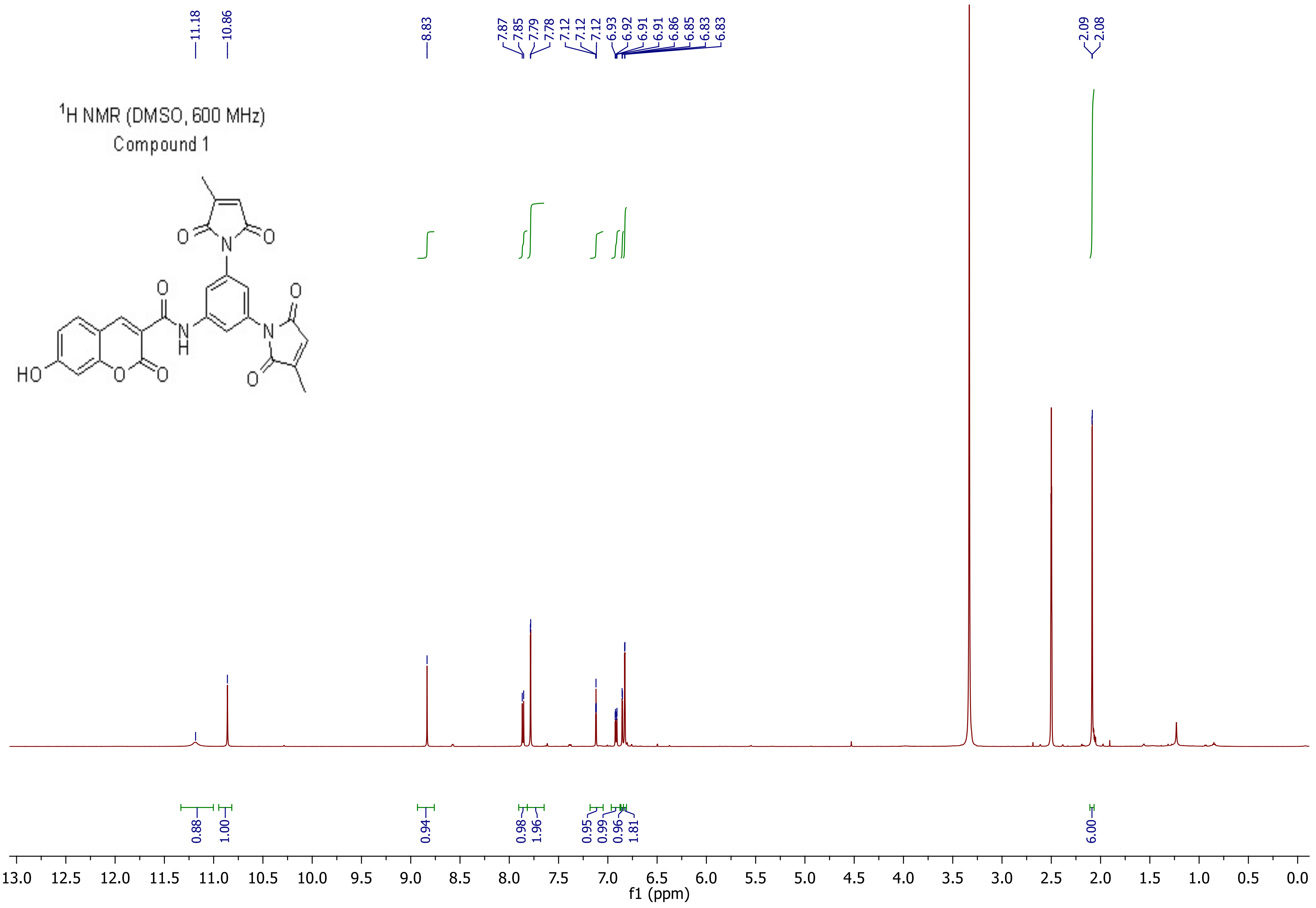

1H NMR (DMSO, 600 MHz)
Compound 1
HO
O
O
O
N
H
N
O
O
N
O
O
11.18
10.86
8.83
7.87
7.85
7.79
7.78
7.12
7.12
7.12
6.93
6.92
6.91
6.91
6.86
6.85
6.83
6.83
2.09
2.08
0.88
1.00
0.94
0.98
1.96
0.95
0.99
0.96
1.81
6.00
13.0 12.5 12.0 11.5 11.0 10.5 10.0 9.5 9.0 8.5 8.0 7.5 7.0 6.5 6.0 5.5 5.0 4.5 4.0 3.5 3.0 2.5 2.0 1.5 1.0 0.5 0.0
f1 (ppm)

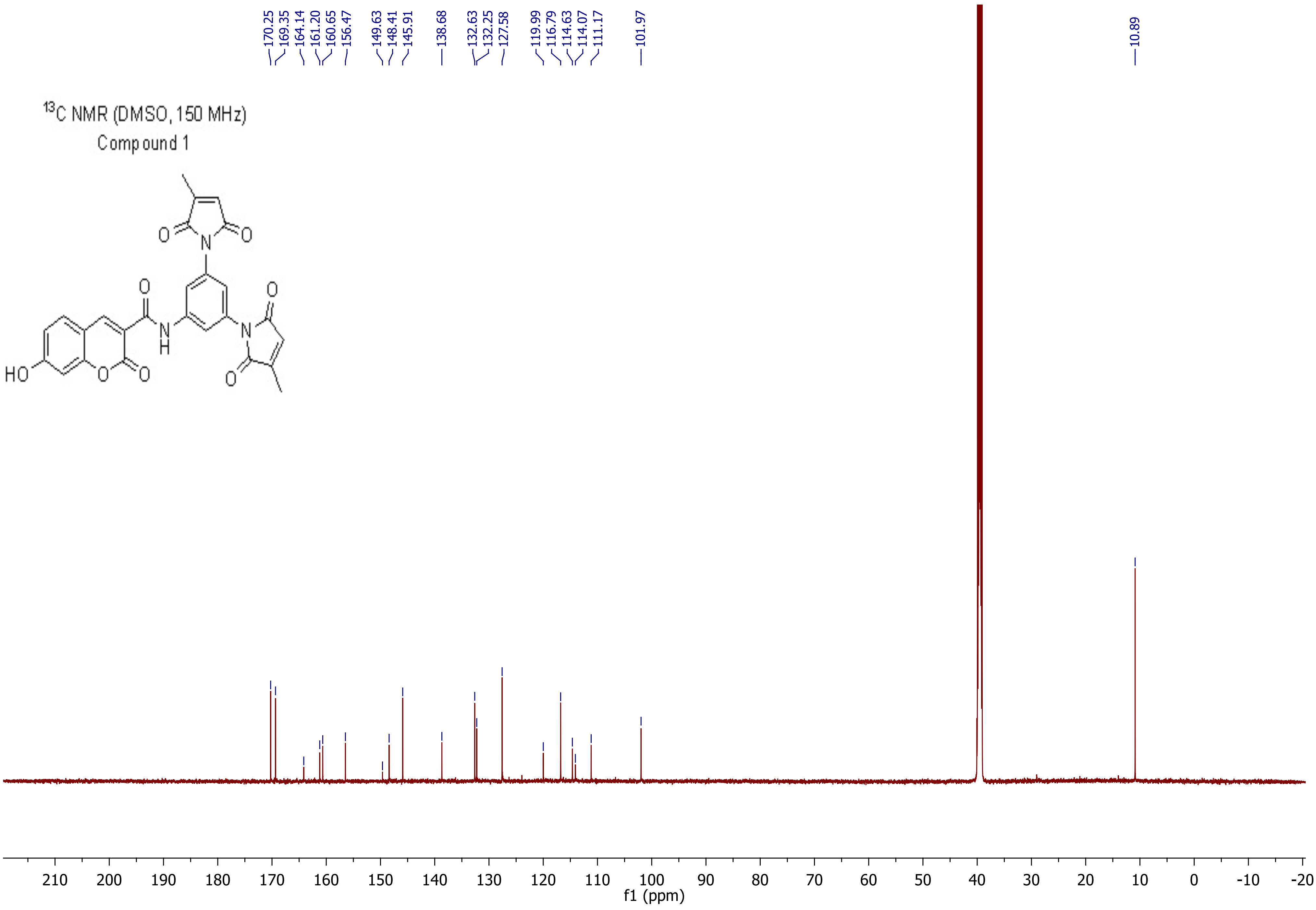

13C NMR (DMSO, 150 MHz)
Compound 1
HO
O
N
H
170.25
169.35
164.14
161.20
160.65
156.47
149.63
148.41
145.91
138.68
132.63
132.25
127.58
119.99
116.79
114.63
114.07
111.17
101.97
10.89
210
200
190
180
170
160
150
140
130
120
110
100
90
80
70
60
50
40
30
20
10
0
-10
-20
f1 (ppm)

^1H NMR ($CDCl_3$, 400 MHz)

Compound 8

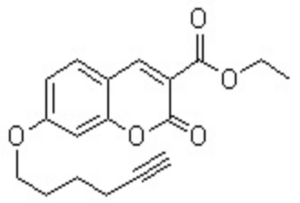

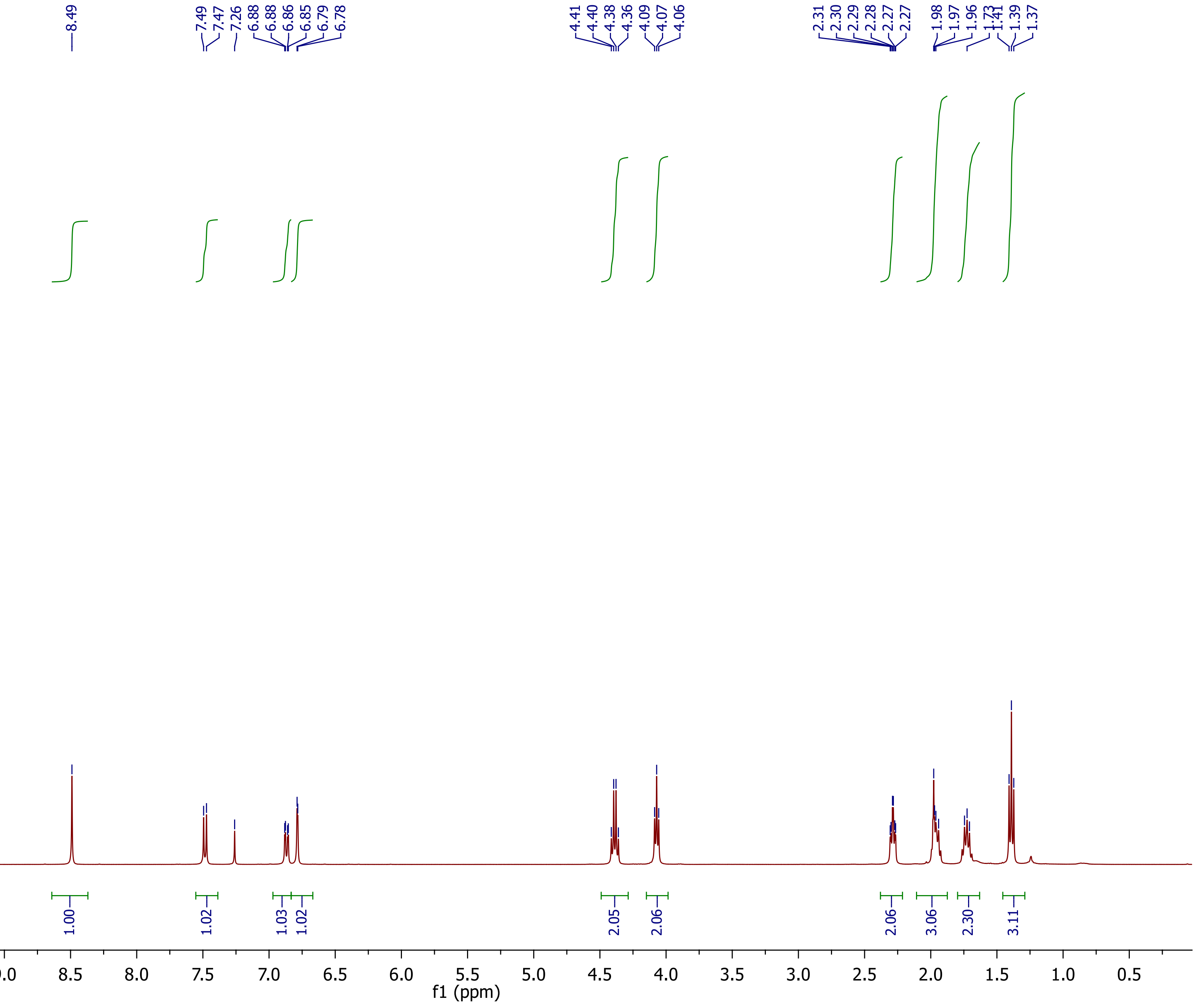

^{13}C NMR ($CDCl_3$, 100 MHz)

Compound 8

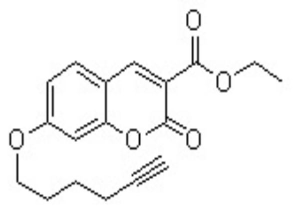

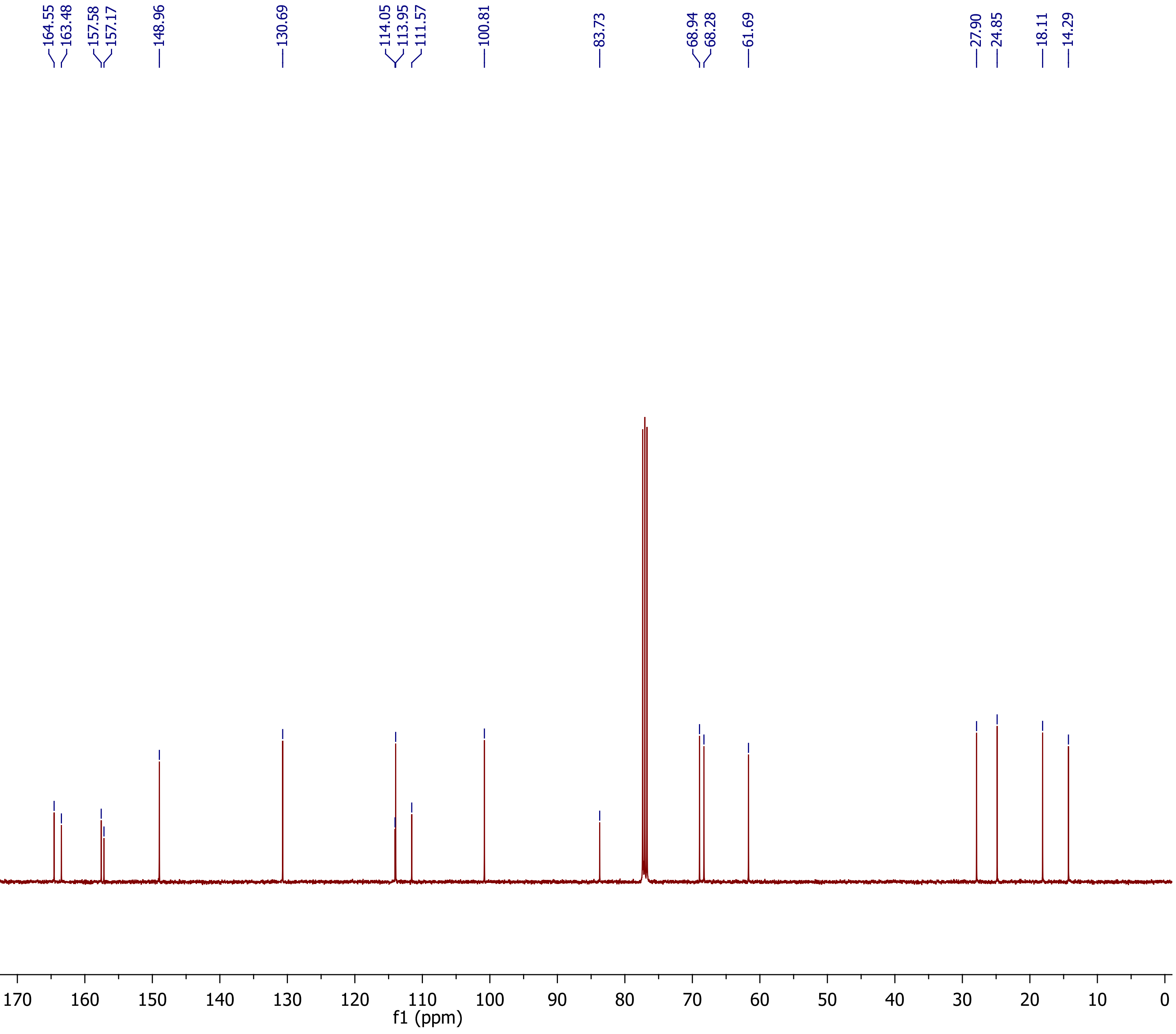

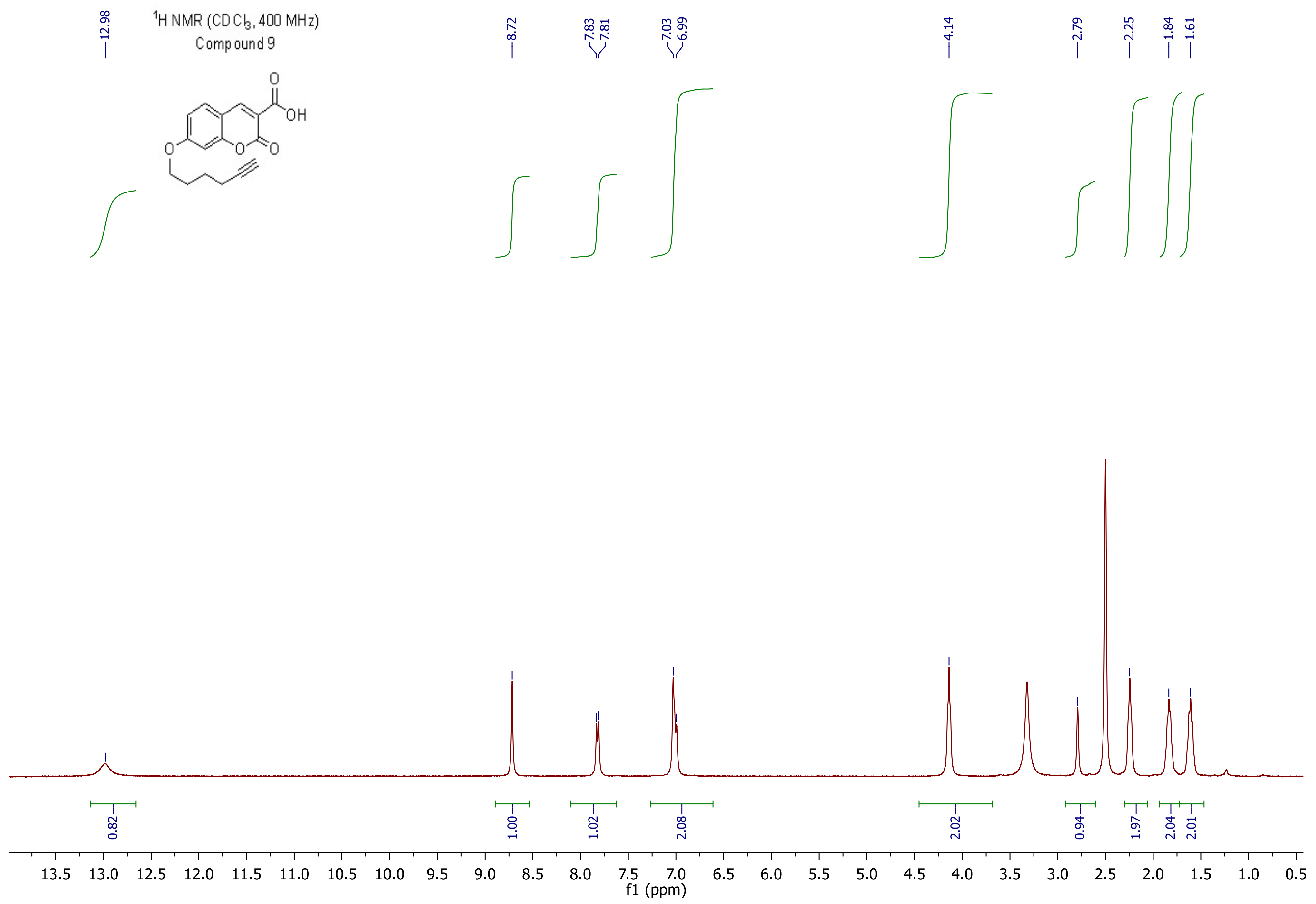

^{1}H NMR (CDCl$_3$, 400 MHz)
Compound 9
O
OH
O
O
O
12.98
8.72
7.83
7.81
7.03
6.99
4.14
2.79
2.25
1.84
1.61
0.82
1.00
1.02
2.08
2.02
0.94
1.97
2.04
2.01
13.5 13.0 12.5 12.0 11.5 11.0 10.5 10.0 9.5 9.0 8.5 8.0 7.5 7.0 6.5 6.0 5.5 5.0 4.5 4.0 3.5 3.0 2.5 2.0 1.5 1.0 0.5
f1 (ppm)

^{13}C NMR ($CDCl_3$, 100 MHz)

Compound 9

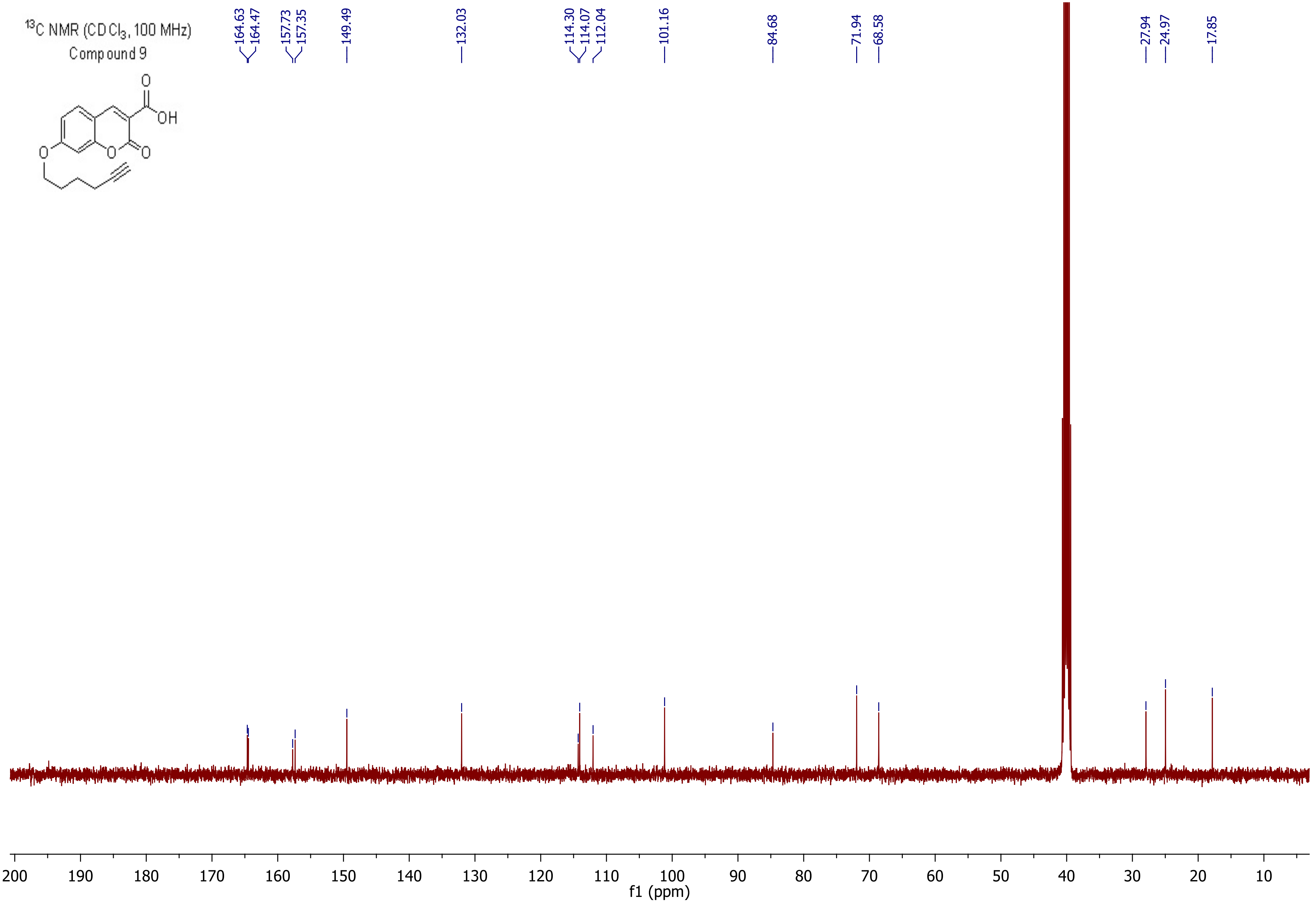

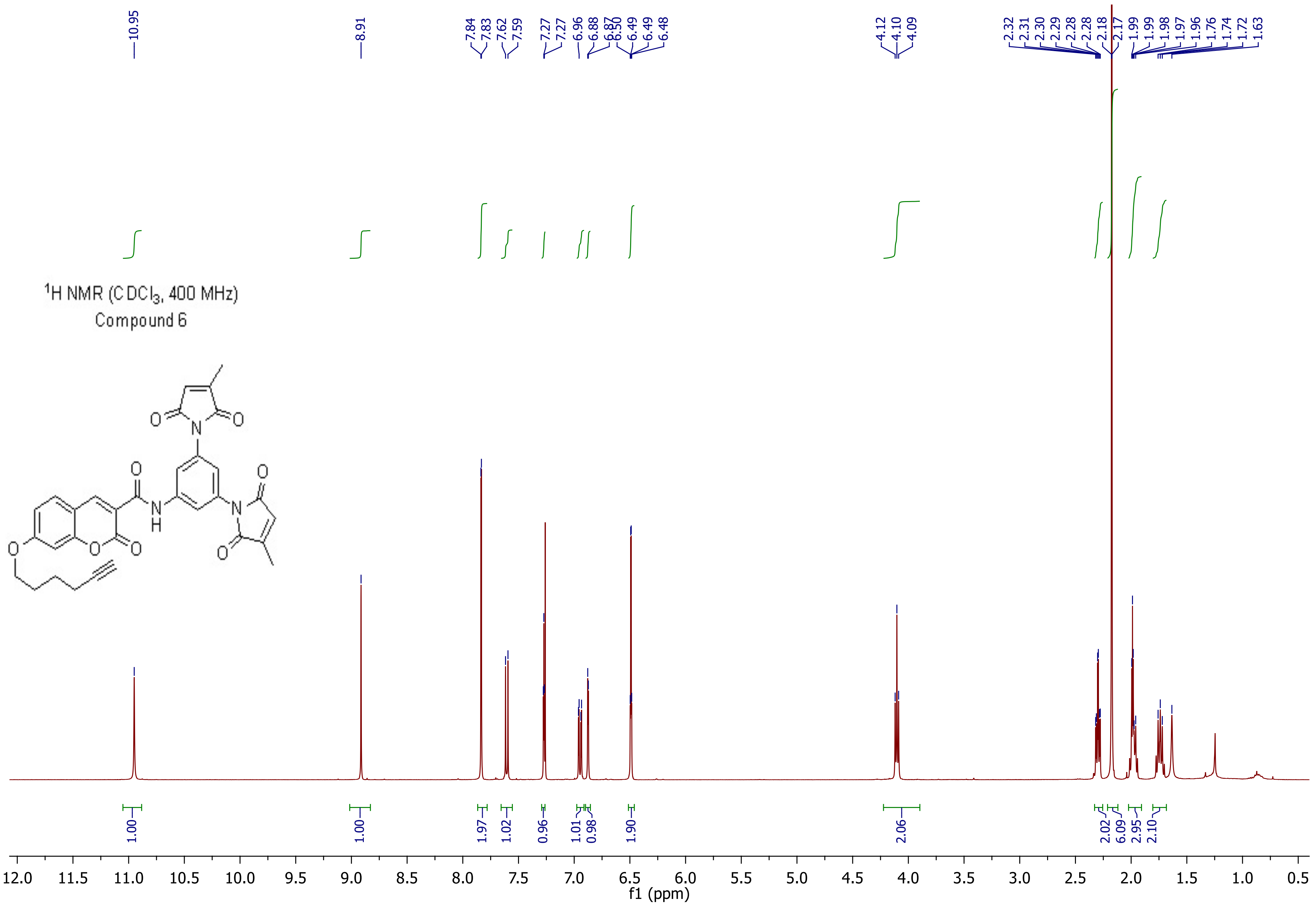

1H NMR (CDCl3, 400 MHz)
Compound 6
10.95
8.91
7.84
7.83
7.62
7.59
7.27
7.27
6.96
6.88
6.87
6.50
6.49
6.49
6.48
4.12
4.10
4.09
2.32
2.31
2.30
2.29
2.28
2.28
2.18
2.17
1.99
1.99
1.98
1.97
1.96
1.76
1.74
1.72
1.63
1.00
1.00
1.97
1.02
0.96
1.01
0.98
1.90
2.06
2.02
6.09
2.95
2.10
12.0
11.5
11.0
10.5
10.0
9.5
9.0
8.5
8.0
7.5
7.0
6.5
6.0
5.5
5.0
4.5
4.0
3.5
3.0
2.5
2.0
1.5
1.0
0.5
f1 (ppm)

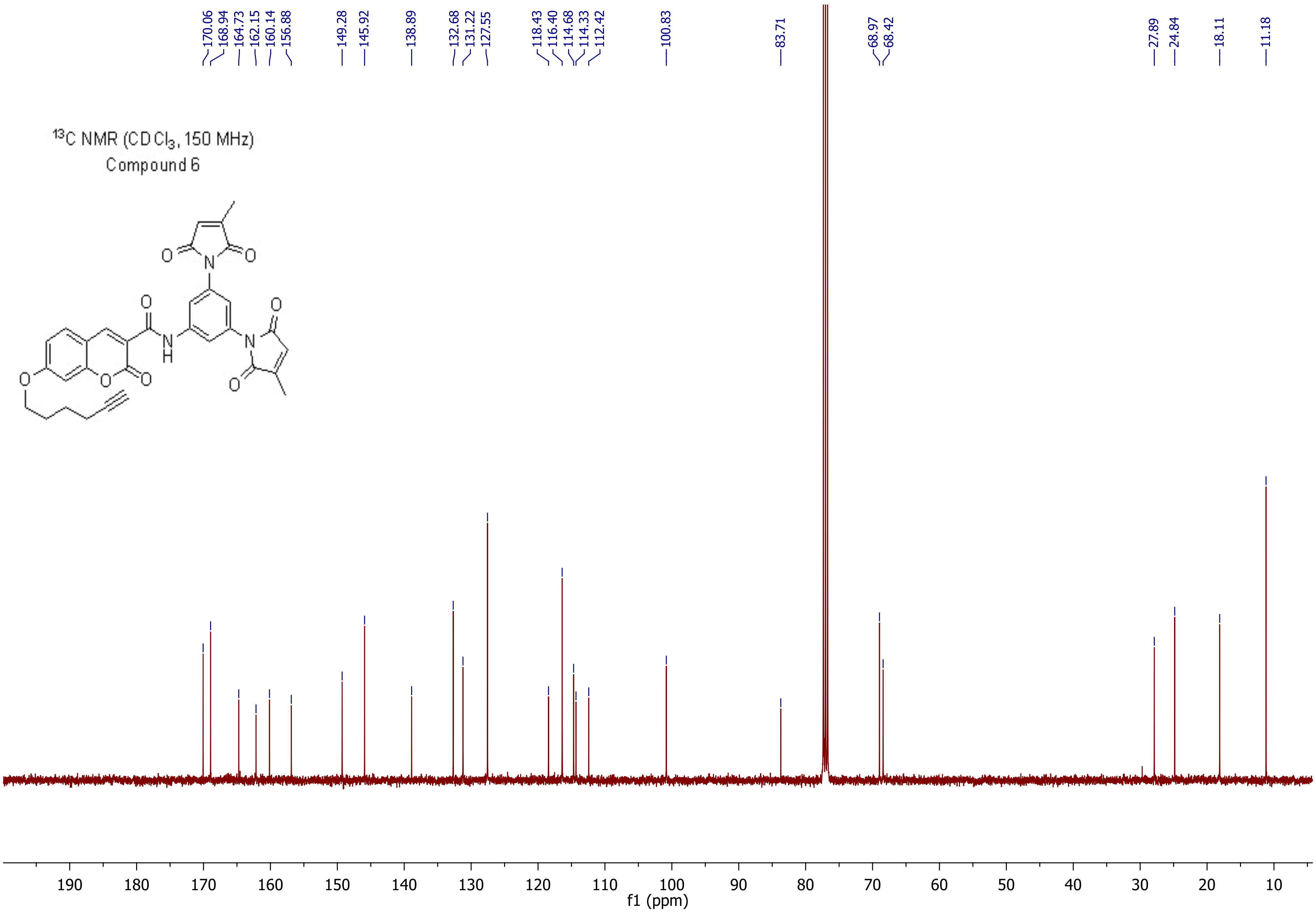

170.06
168.94
164.73
162.15
160.14
156.88
149.28
145.92
138.89
132.68
131.22
127.55
118.43
116.40
114.68
114.33
112.42
100.83
83.71
68.97
68.42
27.89
24.84
18.11
11.18
^{13}C NMR ($CDCl_3$, 150 MHz)
Compound 6
190
180
170
160
150
140
130
120
110
100
90
80
70
60
50
40
30
20
10
f1 (ppm)

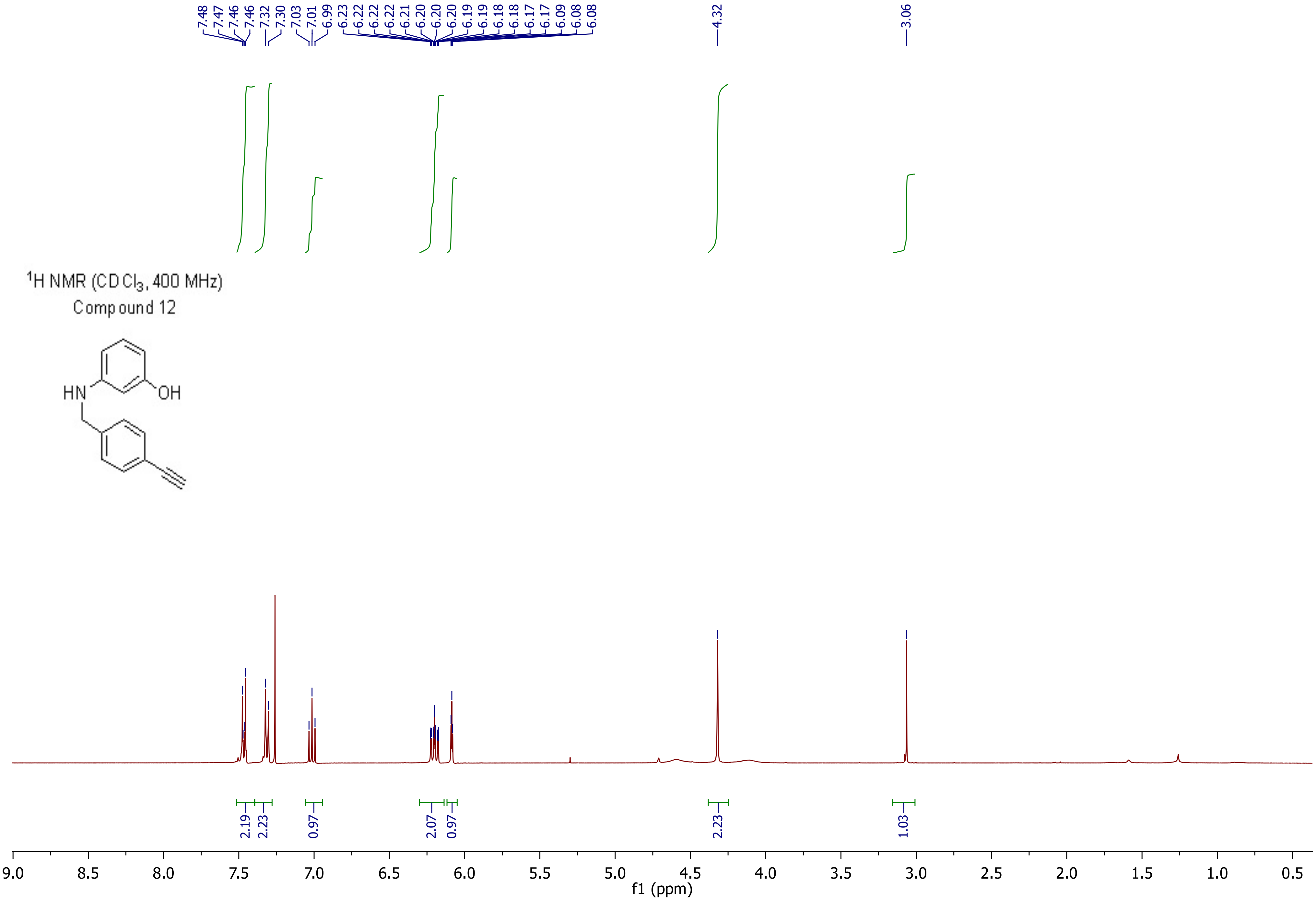

^{1}H NMR (CDCl$_3$, 400 MHz)
Compound 12
HN
OH
7.48
7.47
7.46
7.46
7.32
7.30
7.03
7.01
6.99
6.23
6.22
6.22
6.22
6.21
6.20
6.20
6.20
6.19
6.19
6.18
6.18
6.17
6.17
6.09
6.08
6.08
4.32
3.06
2.19
2.23
0.97
2.07
0.97
2.23
1.03
9.0
8.5
8.0
7.5
7.0
6.5
6.0
5.5
5.0
4.5
4.0
3.5
3.0
2.5
2.0
1.5
1.0
0.5
f1 (ppm)

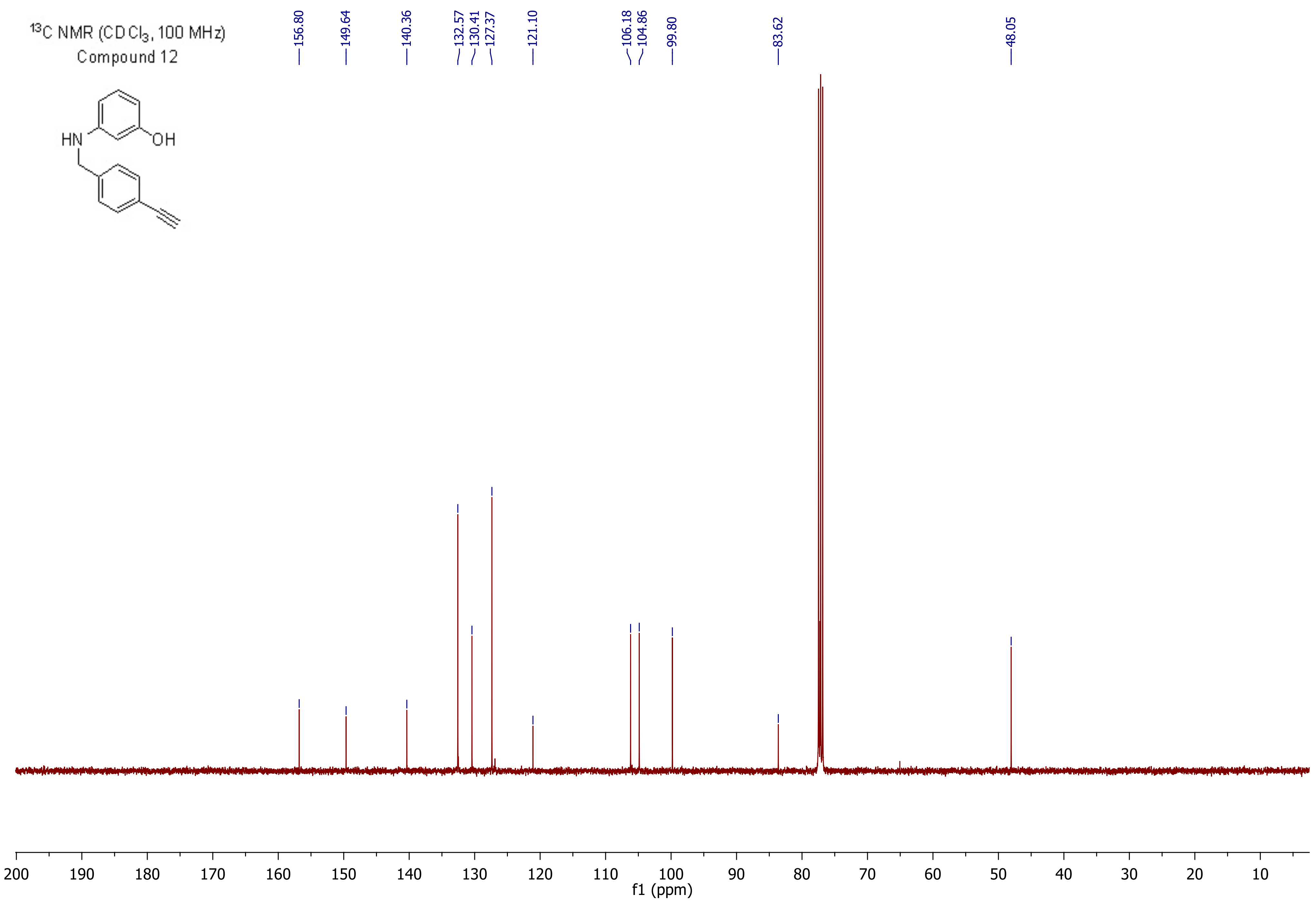

^{13}C NMR (CDCl$_3$, 100 MHz)
Compound 12
HN
OH
156.80
149.64
140.36
132.57
130.41
127.37
121.10
106.18
104.86
99.80
83.62
48.05
200 190 180 170 160 150 140 130 120 110 100 90 80 70 60 50 40 30 20 10
f1 (ppm)

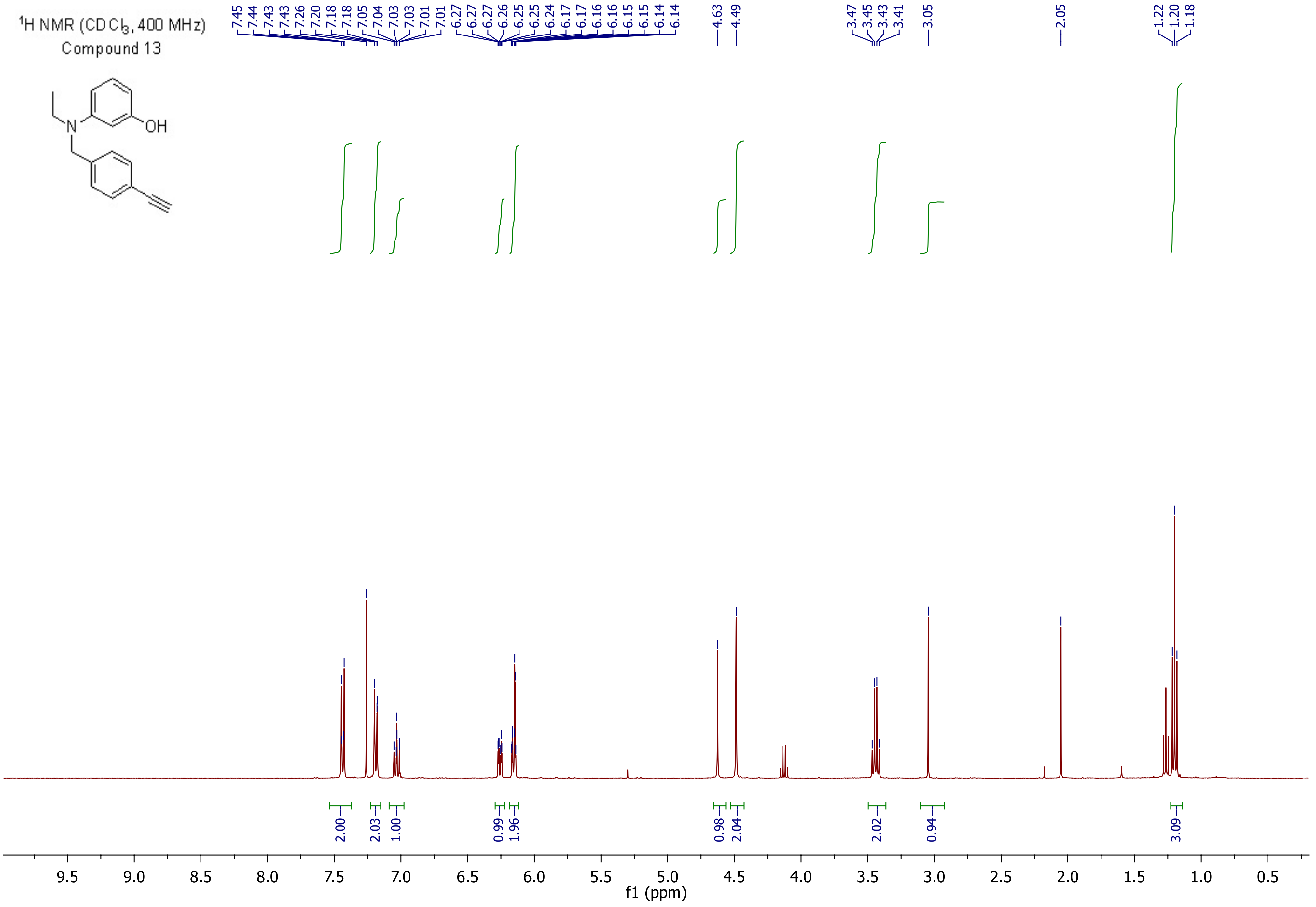

1H NMR (CDCl3, 400 MHz)
Compound 13
OH
N
7.45 7.44 7.43 7.43 7.26 7.20 7.18 7.18 7.05 7.04 7.03 7.03 7.01 7.01 6.27 6.27 6.27 6.26 6.25 6.25 6.24 6.17 6.17 6.16 6.16 6.15 6.15 6.14 6.14
4.63 4.49
3.47 3.45 3.43 3.41 3.05
2.05
1.22 1.20 1.18
2.00 2.03 1.00 0.99 1.96 0.98 2.04 2.02 0.94 3.09
9.5 9.0 8.5 8.0 7.5 7.0 6.5 6.0 5.5 5.0 4.5 4.0 3.5 3.0 2.5 2.0 1.5 1.0 0.5
f1 (ppm)

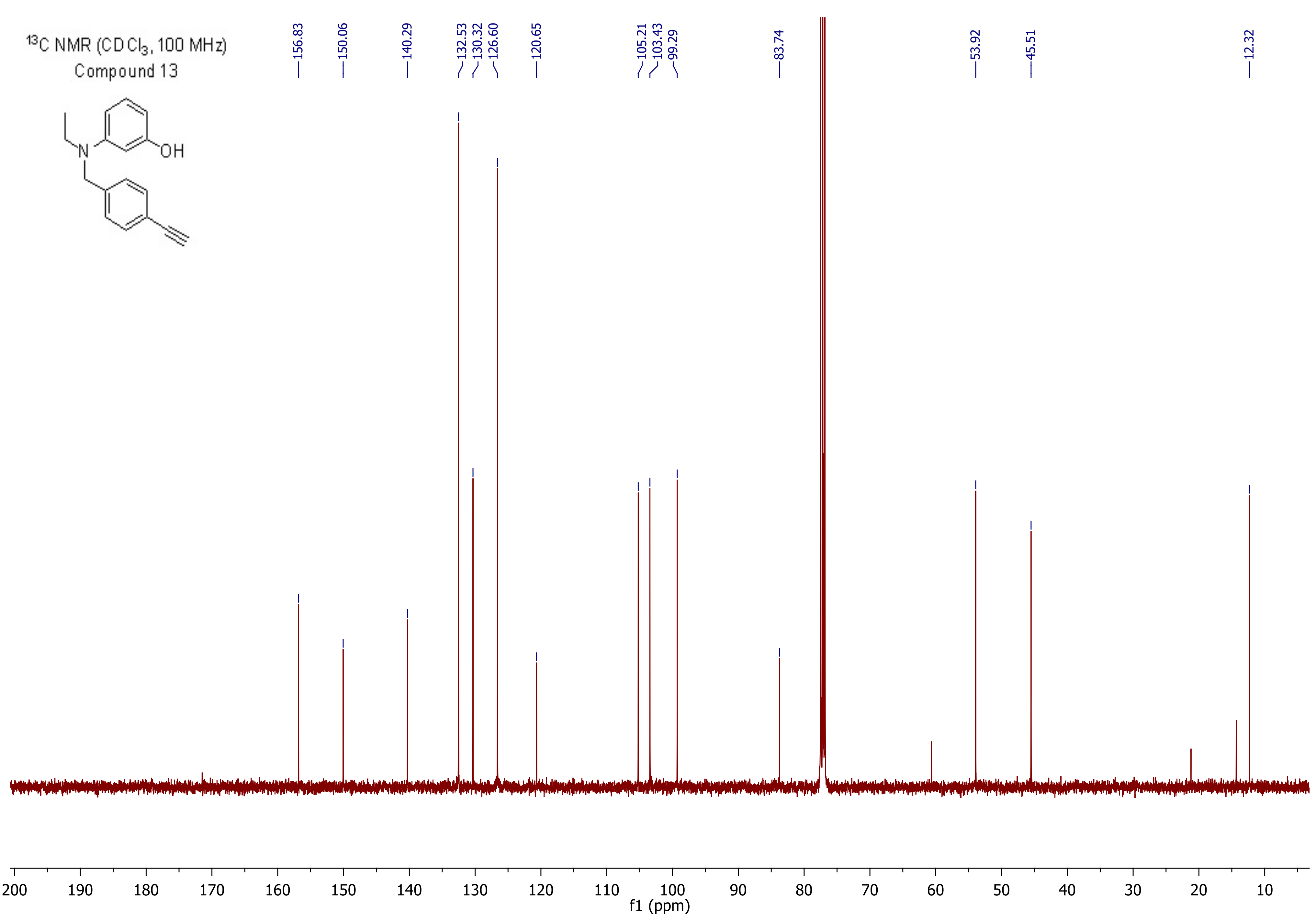

13C NMR (CDCl3, 100 MHz)
Compound 13
OH
N
156.83
150.06
140.29
132.53
130.32
126.60
120.65
105.21
103.43
99.29
83.74
53.92
45.51
12.32
200
190
180
170
160
150
140
130
120
110
100
90
80
70
60
50
40
30
20
10
f1 (ppm)

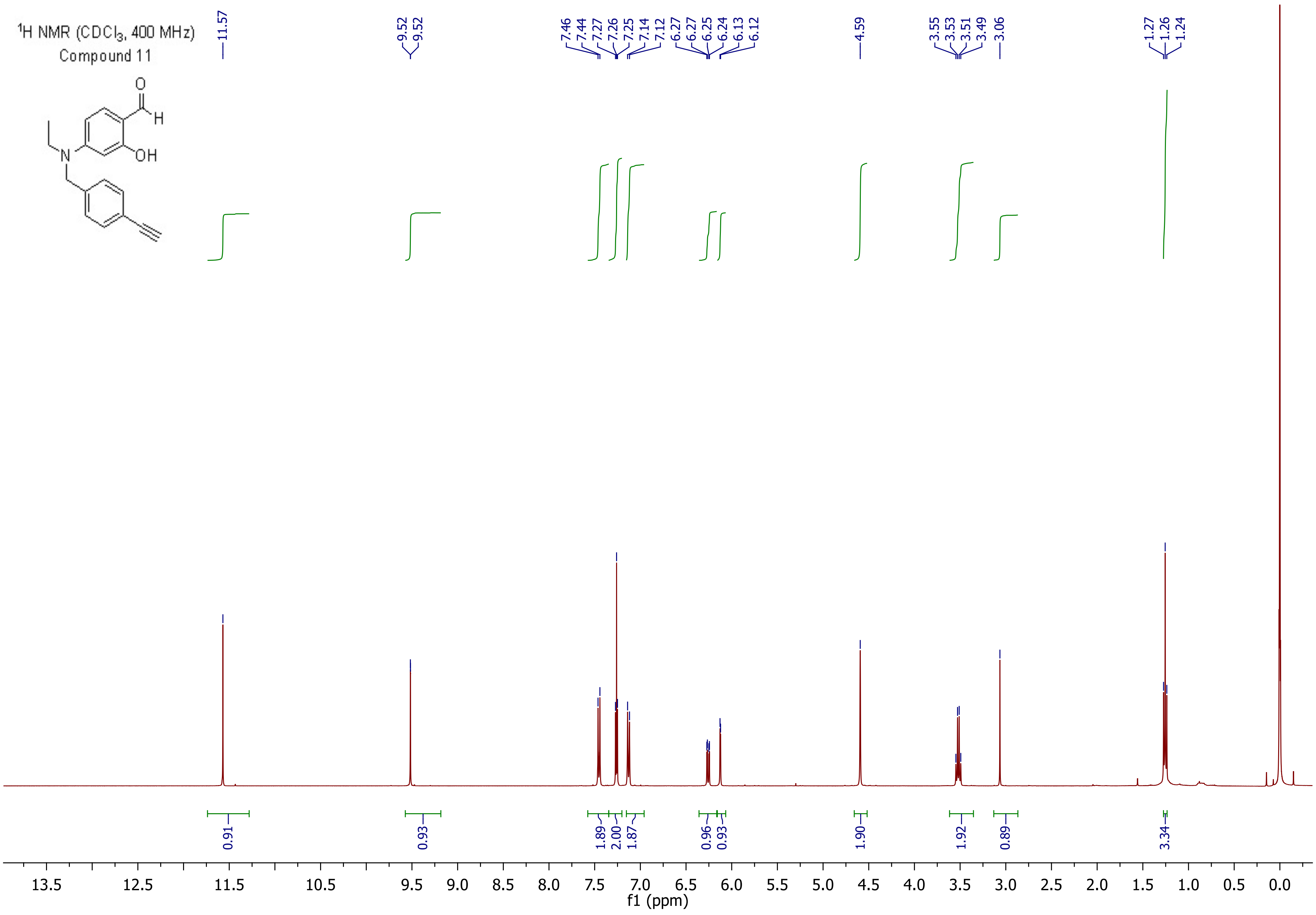

^{1}H NMR (CDCl$_3$, 400 MHz)
Compound 11
O
H
OH
N
11.57
9.52
9.52
7.46
7.44
7.27
7.26
7.25
7.14
7.12
6.27
6.27
6.25
6.24
6.13
6.12
4.59
3.55
3.53
3.51
3.49
3.06
1.27
1.26
1.24
0.91
0.93
1.89
2.00
1.87
0.96
0.93
1.90
1.92
0.89
3.34
13.5
12.5
11.5
10.5
9.5
9.0
8.5
8.0
7.5
7.0
6.5
6.0
5.5
5.0
4.5
4.0
3.5
3.0
2.5
2.0
1.5
1.0
0.5
0.0
f1 (ppm)

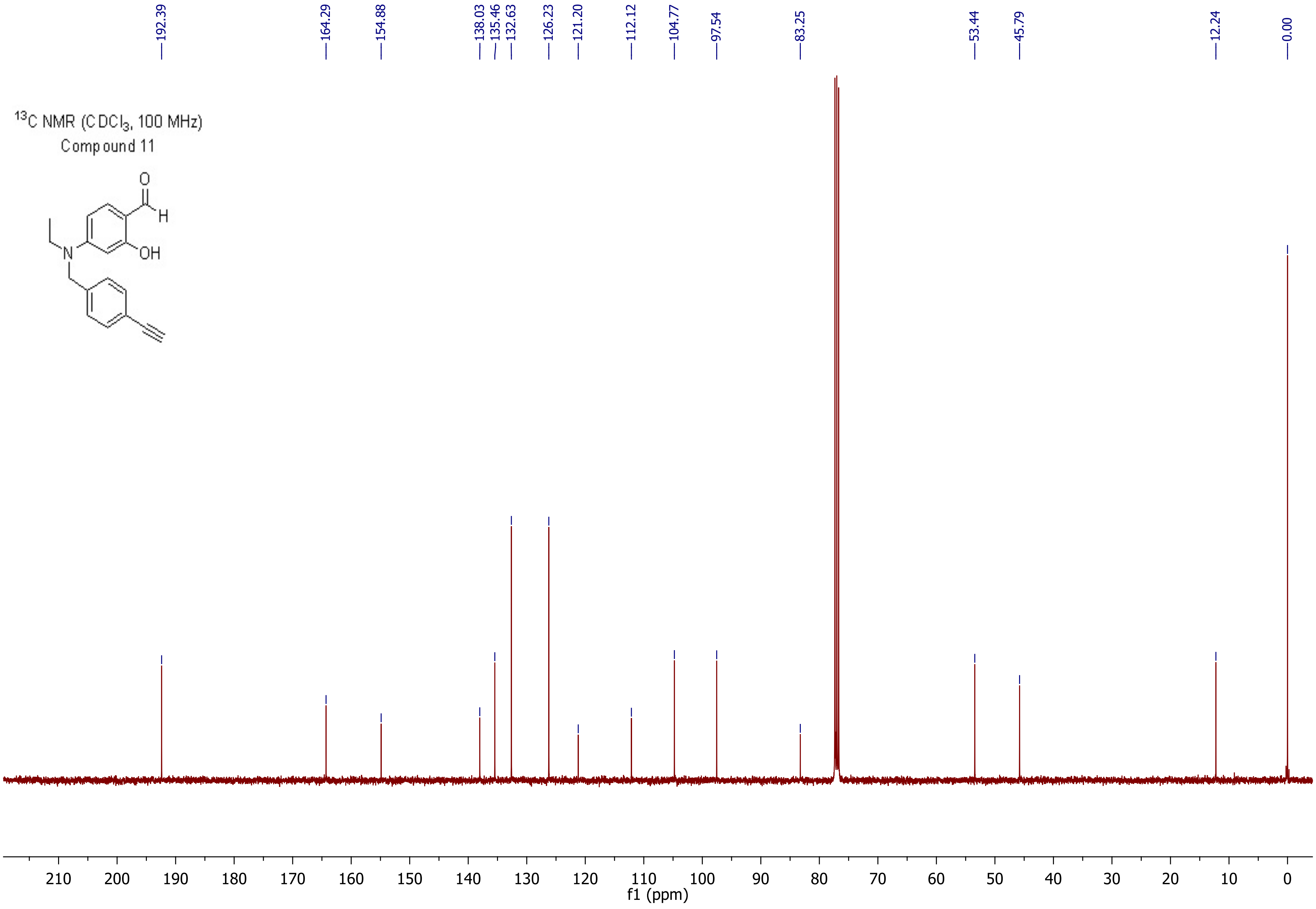

192.39
164.29
154.88
138.03
135.46
132.63
126.23
121.20
112.12
104.77
97.54
83.25
53.44
45.79
12.24
0.00
^{13}C NMR ($CDCl_3$, 100 MHz)
Compound 11
O
H
OH
N
210 200 190 180 170 160 150 140 130 120 110 100 90 80 70 60 50 40 30 20 10 0
f1 (ppm)

^1H NMR ($CDCl_3$, 400 MHz)
Compound 14

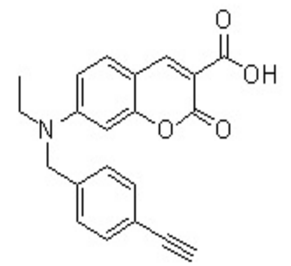

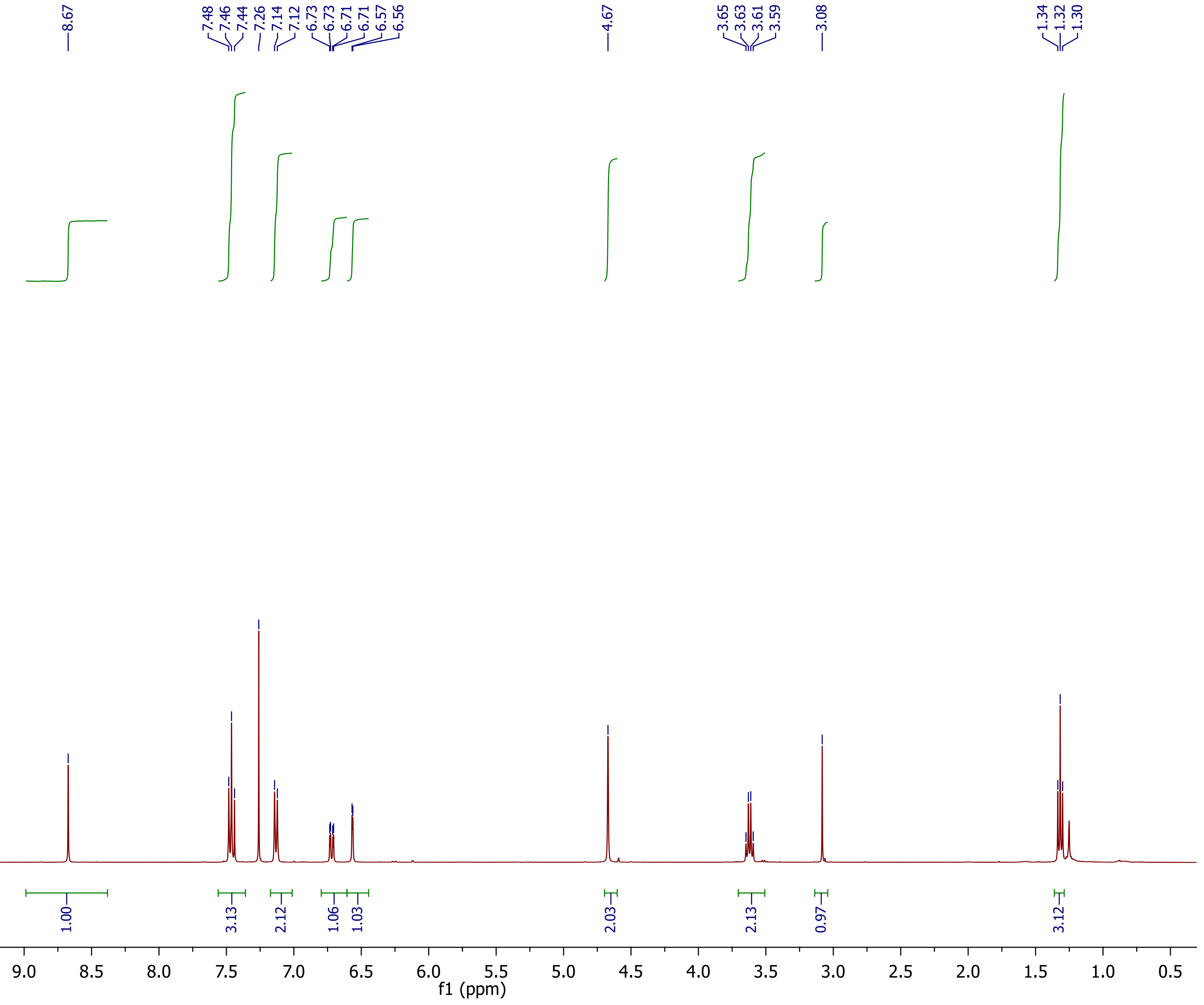

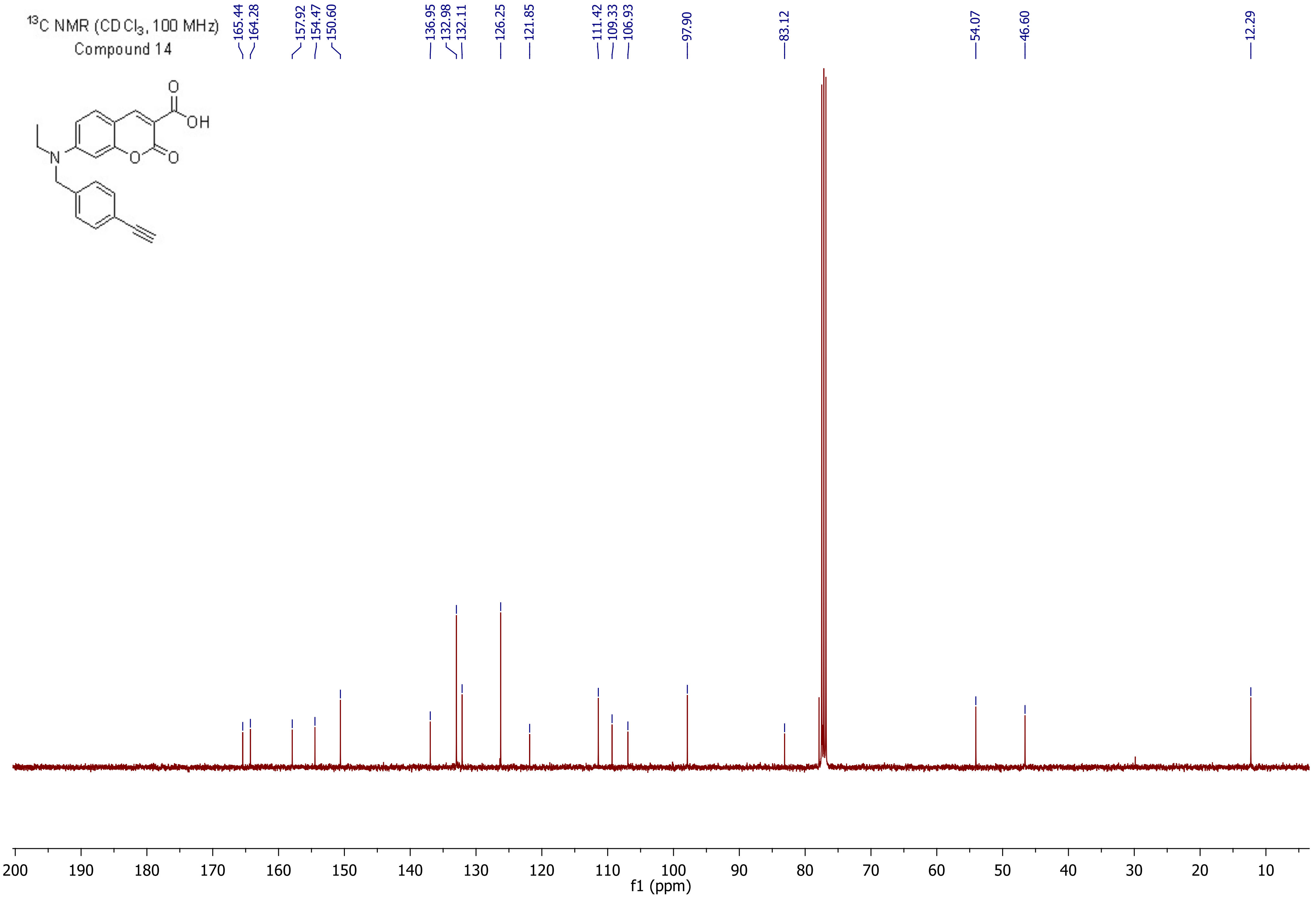

^{13}C NMR ($CDCl_3$, 100 MHz)
Compound 14
O
OH
N
O
O
165.44
164.28
157.92
154.47
150.60
136.95
132.98
132.11
126.25
121.85
111.42
109.33
106.93
97.90
83.12
54.07
46.60
12.29
200 190 180 170 160 150 140 130 120 110 100 90 80 70 60 50 40 30 20 10
f1 (ppm)

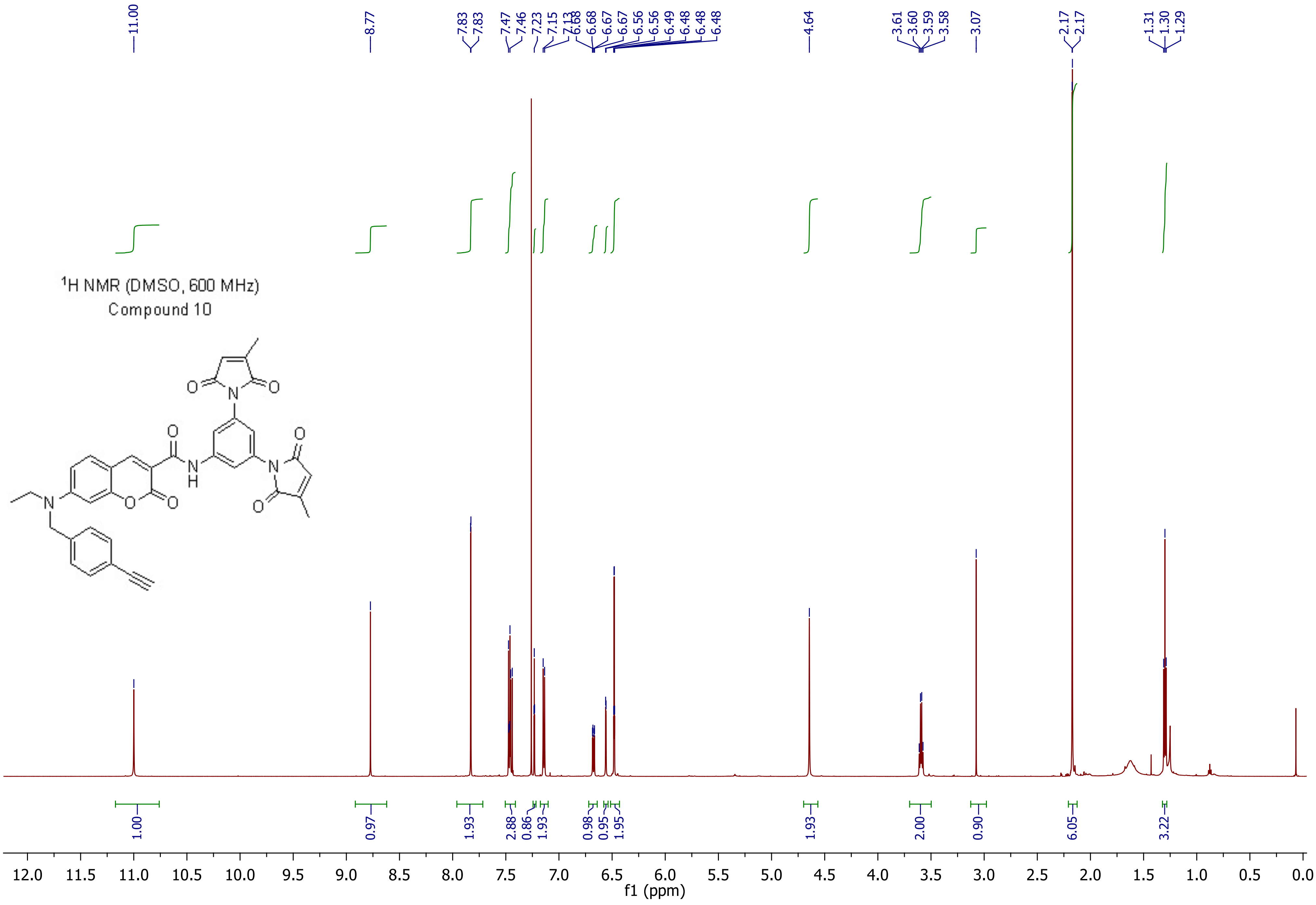

1H NMR (DMSO, 600 MHz)
Compound 10
11.00
8.77
7.83
7.83
7.47
7.46
7.23
7.15
7.13
6.68
6.68
6.67
6.67
6.56
6.56
6.49
6.48
6.48
6.48
4.64
3.61
3.60
3.59
3.58
3.07
2.17
2.17
1.31
1.30
1.29
1.00
0.97
1.93
2.88
0.86
1.93
0.98
0.95
1.95
1.93
2.00
0.90
6.05
3.22
f1 (ppm)

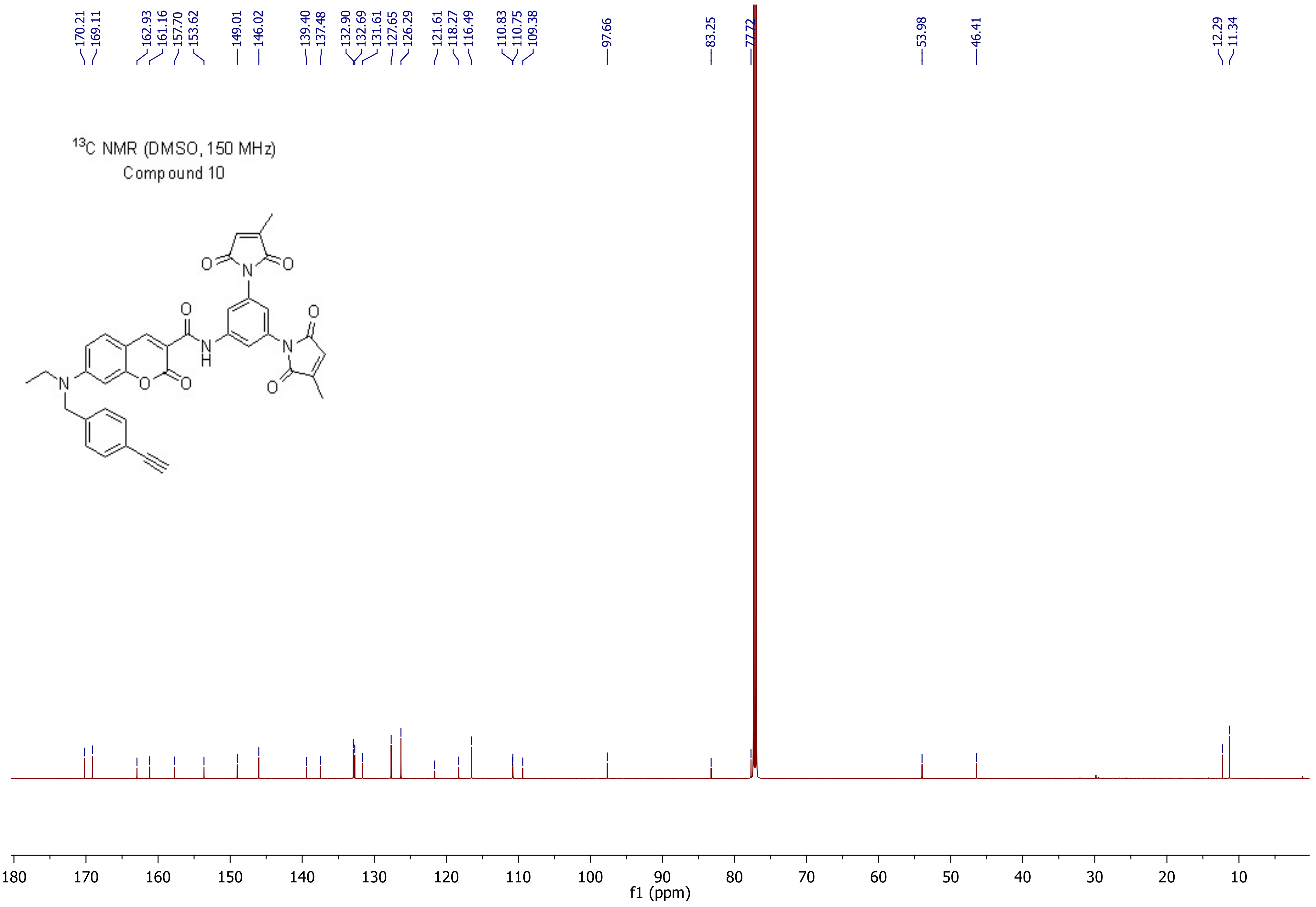

170.21
169.11
162.93
161.16
157.70
153.62
149.01
146.02
139.40
137.48
132.90
132.69
131.61
127.65
126.29
121.61
118.27
116.49
110.83
110.75
109.38
97.66
83.25
77.72
53.98
46.41
12.29
11.34
^{13}C NMR (DMSO, 150 MHz)
Compound 10
180 170 160 150 140 130 120 110 100 90 80 70 60 50 40 30 20 10
f1 (ppm)

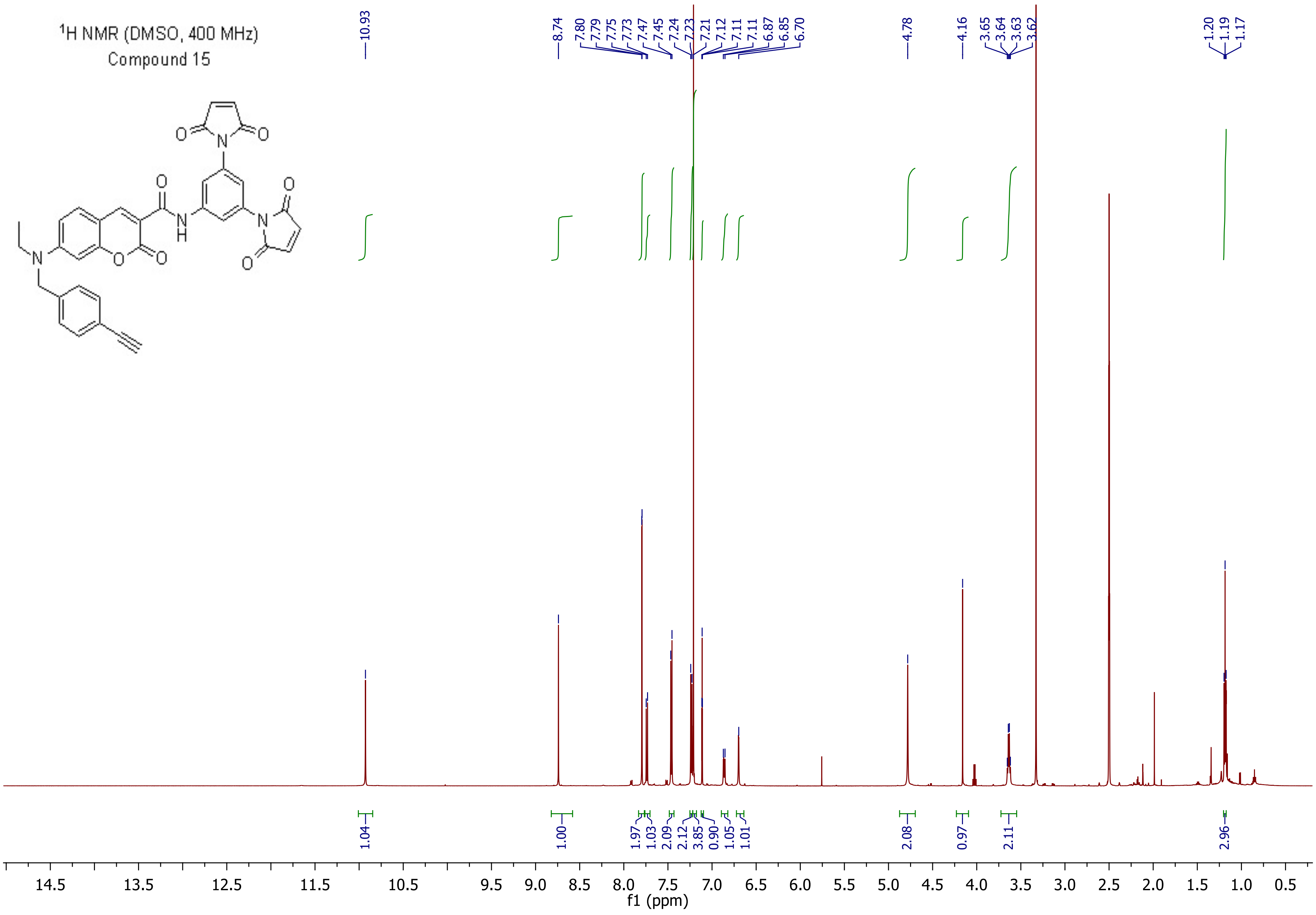

^{1}H NMR (DMSO, 400 MHz)
Compound 15
10.93
8.74
7.80
7.79
7.75
7.73
7.47
7.45
7.24
7.23
7.21
7.12
7.11
7.11
6.87
6.85
6.70
4.78
4.16
3.65
3.64
3.63
3.62
1.20
1.19
1.17
1.04
1.00
1.97
1.03
2.09
2.12
3.85
0.90
1.05
1.01
2.08
0.97
2.11
2.96
14.5
13.5
12.5
11.5
10.5
9.5
9.0
8.5
8.0
7.5
7.0
6.5
6.0
5.5
5.0
4.5
4.0
3.5
3.0
2.5
2.0
1.5
1.0
0.5
f1 (ppm)

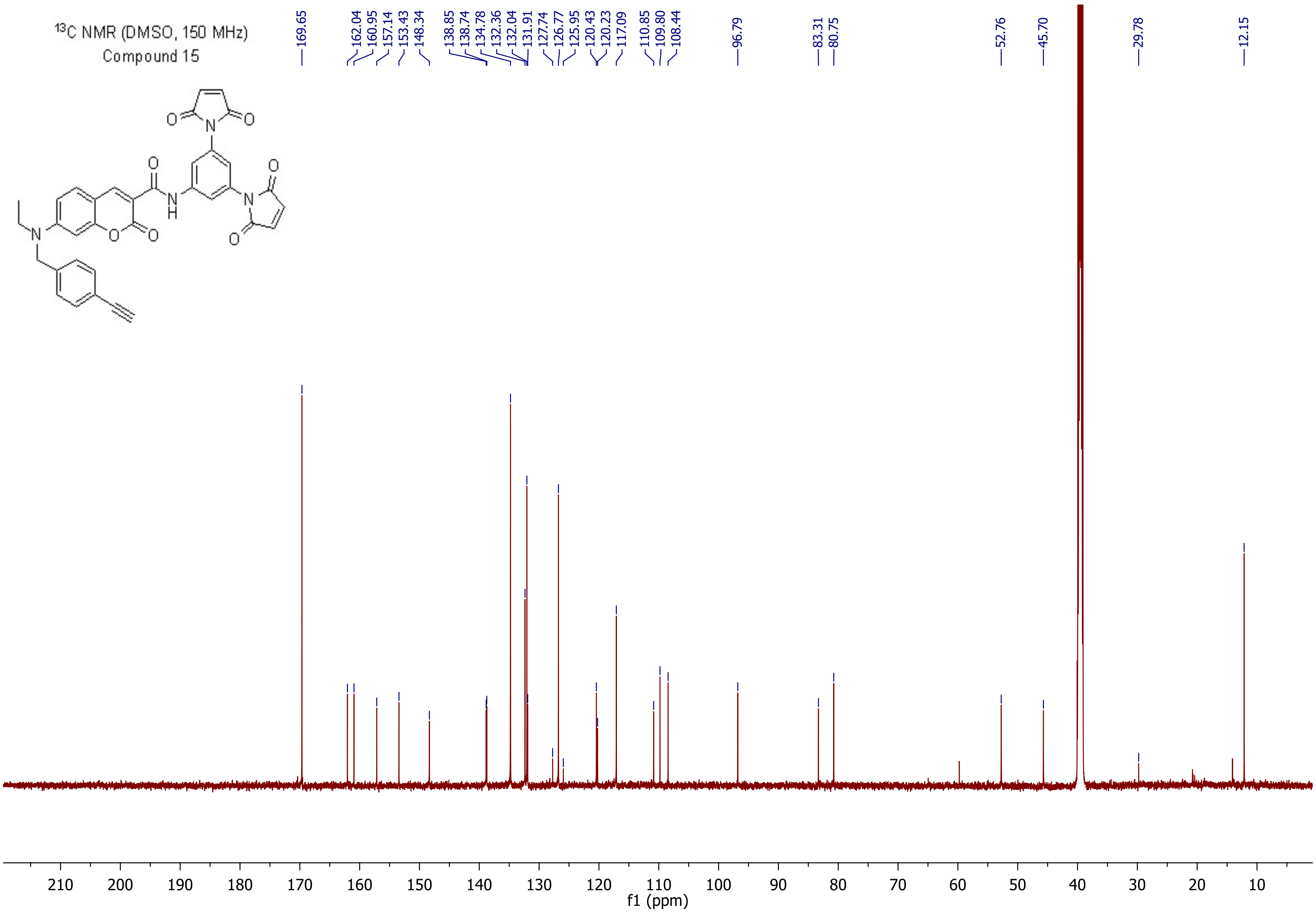

13C NMR (DMSO, 150 MHz)
Compound 15
169.65
162.04
160.95
157.14
153.43
148.34
138.85
138.74
134.78
132.36
132.04
131.91
127.74
126.77
125.95
120.43
120.23
117.09
110.85
109.80
108.44
96.79
83.31
80.75
52.76
45.70
29.78
12.15
210
200
190
180
170
160
150
140
130
120
110
100
90
80
70
60
50
40
30
20
10
f1 (ppm)

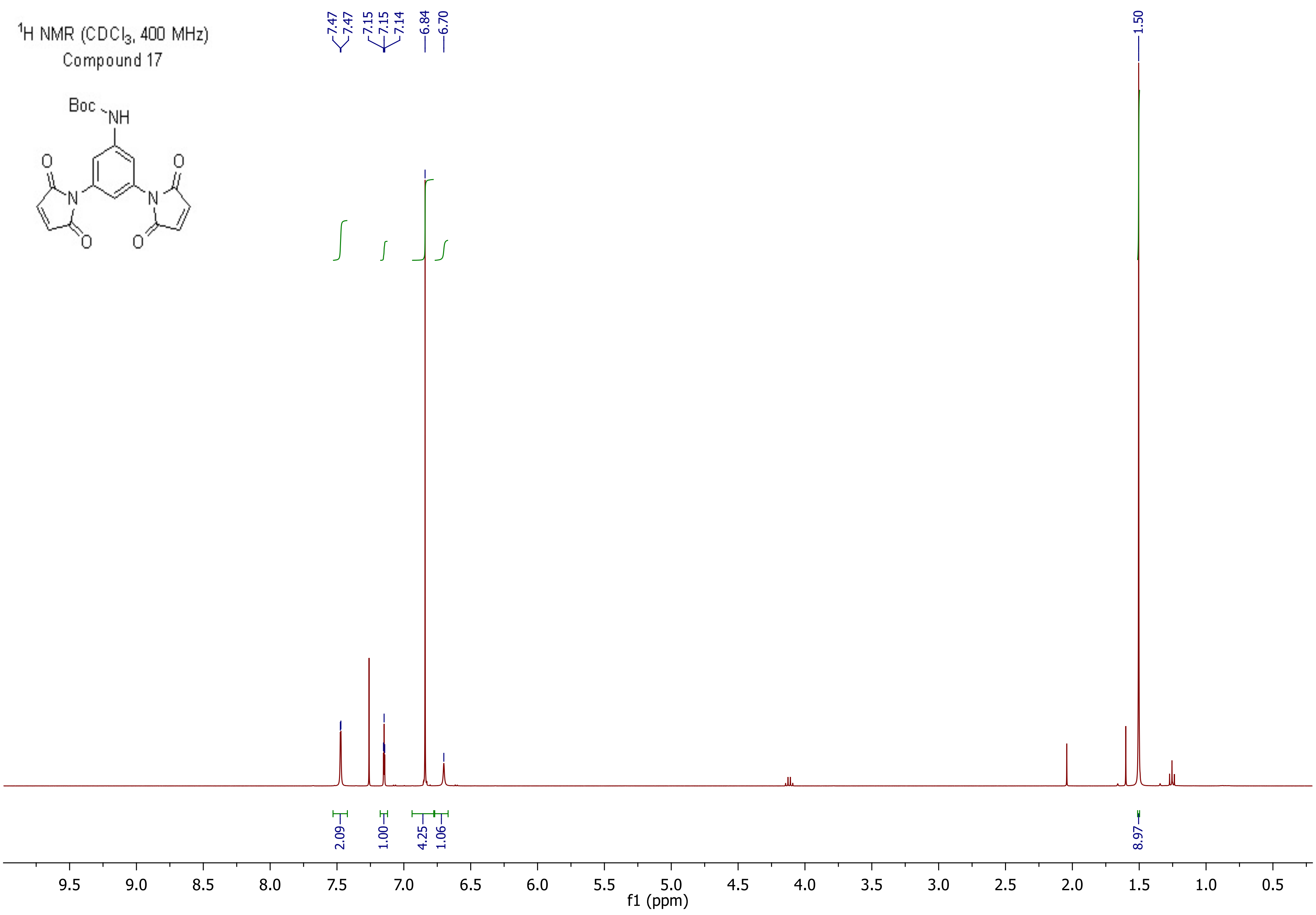

^1H NMR ($CDCl_3$, 400 MHz)
Compound 17
Boc
NH
O
N
7.47
7.47
7.15
7.15
7.14
6.84
6.70
1.50
2.09
1.00
4.25
1.06
8.97
9.5
9.0
8.5
8.0
7.5
7.0
6.5
6.0
5.5
5.0
4.5
4.0
3.5
3.0
2.5
2.0
1.5
1.0
0.5
f1 (ppm)

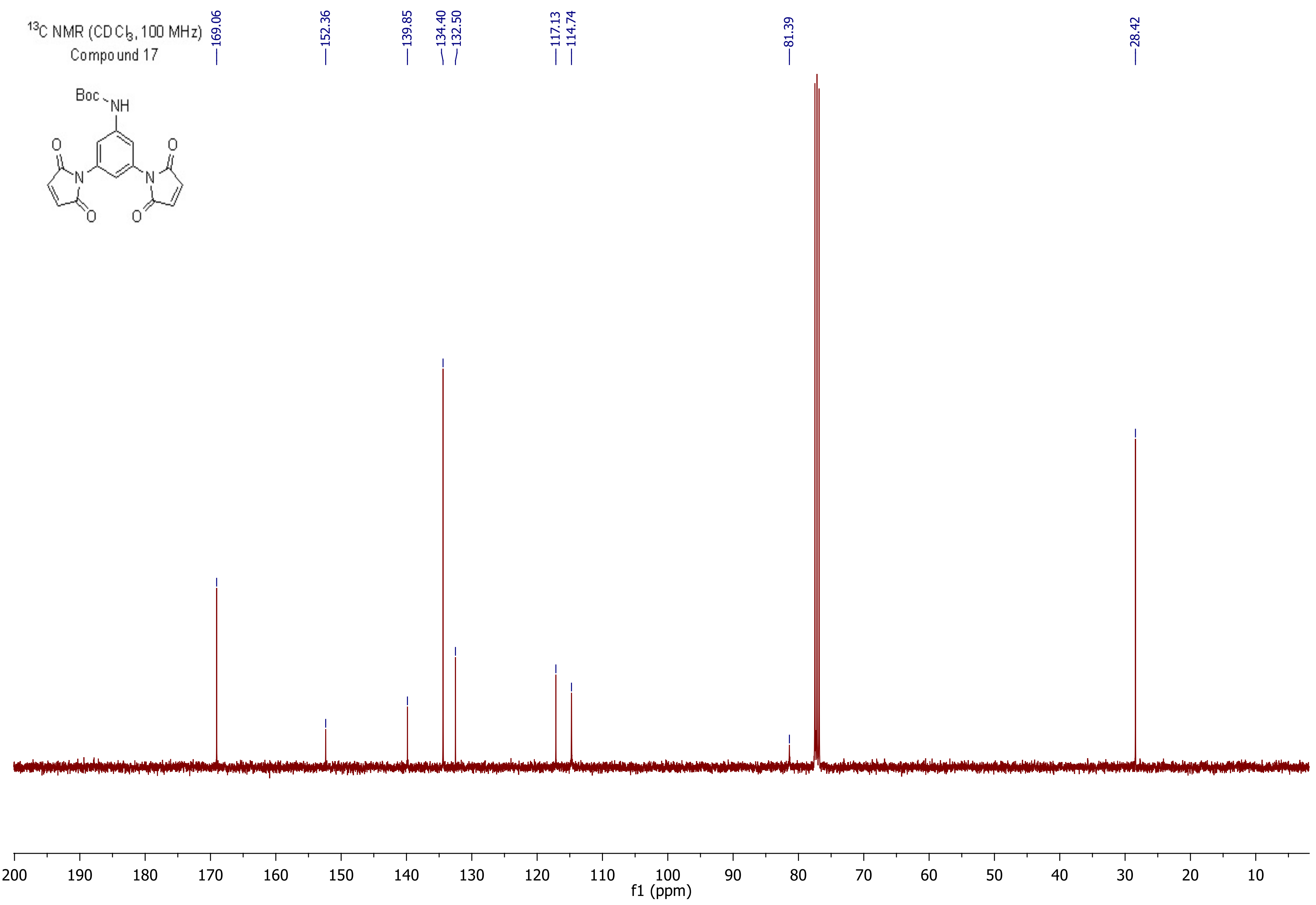

^{13}C NMR (CDCl$_3$, 100 MHz)
Compound 17
Boc
NH
O
N
169.06
152.36
139.85
134.40
132.50
117.13
114.74
81.39
28.42
200
190
180
170
160
150
140
130
120
110
100
90
80
70
60
50
40
30
20
10
f1 (ppm)

^{1}H NMR (DMSO, 400 MHz)
Compound 4

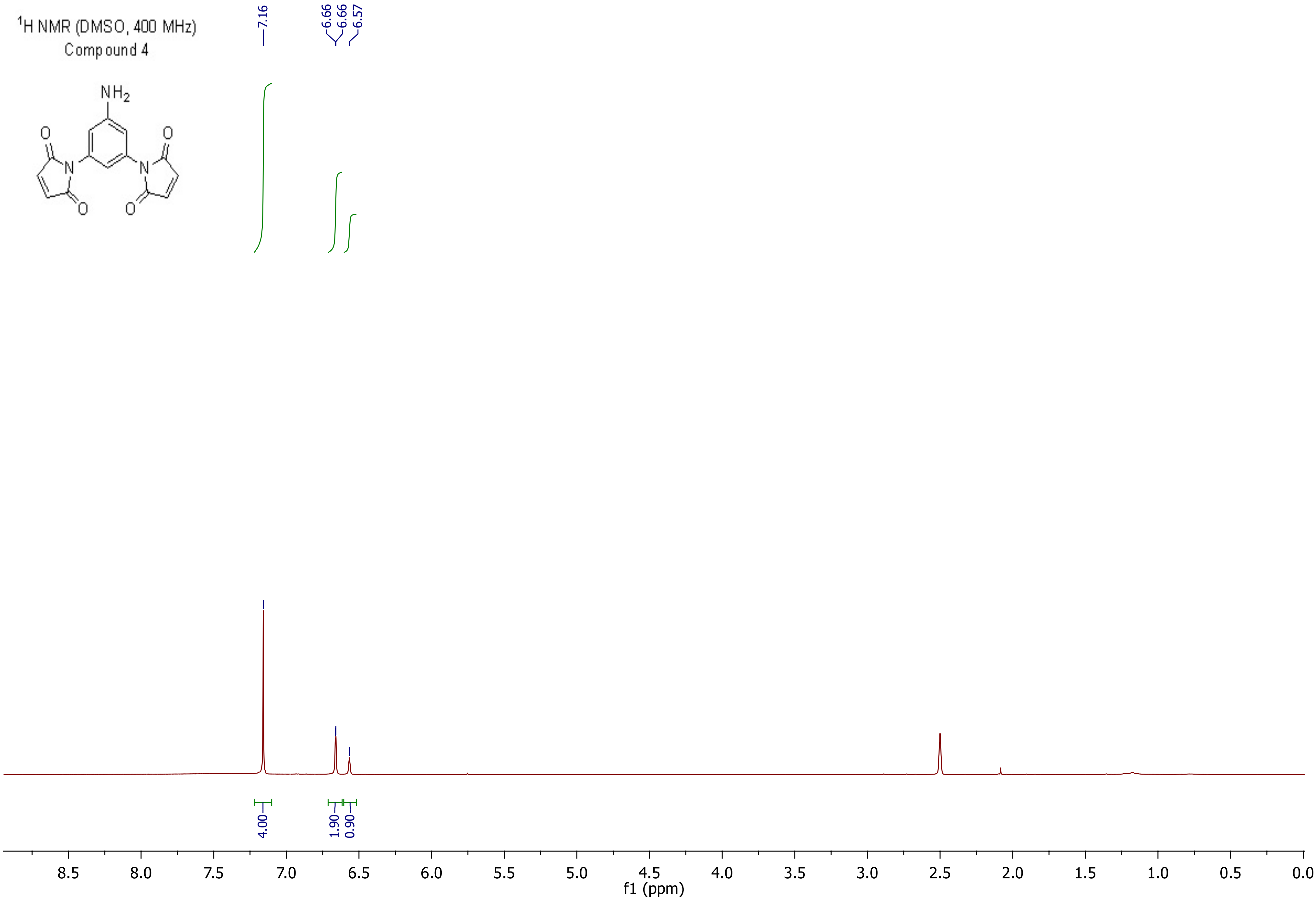

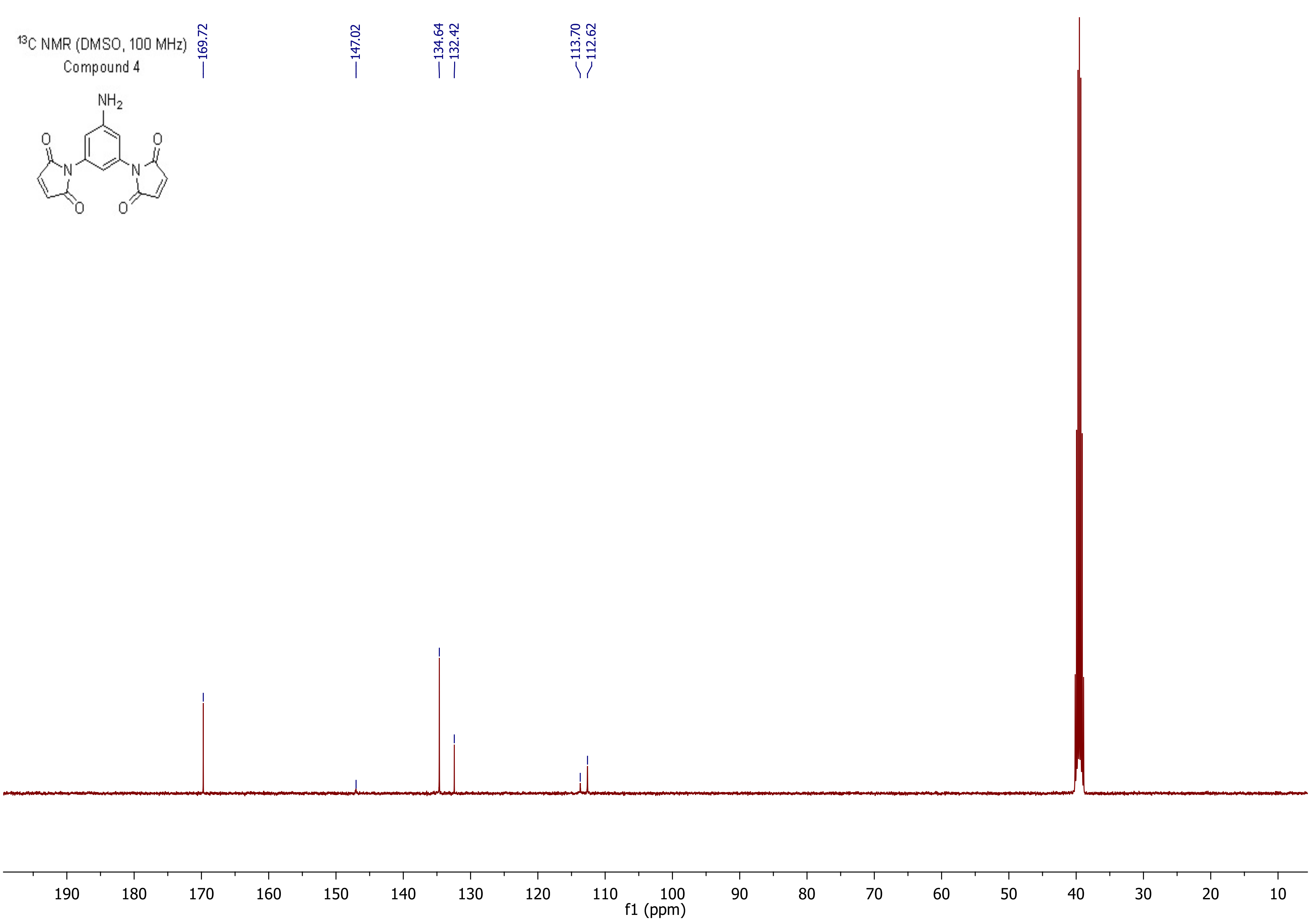

^{13}C NMR (DMSO, 100 MHz)
Compound 4
NH_2
O
N
169.72
147.02
134.64
132.42
113.70
112.62
190
180
170
160
150
140
130
120
110
100
90
80
70
60
50
40
30
20
10
f1 (ppm)
